# Subventricular zone involvement in Glioblastoma – A proteomic evaluation and clinicoradiological correlation

**DOI:** 10.1038/s41598-017-01202-8

**Published:** 2017-05-03

**Authors:** Kishore Gollapalli, Saicharan Ghantasala, Sachendra Kumar, Rajneesh Srivastava, Srikanth Rapole, Aliasgar Moiyadi, Sridhar Epari, Sanjeeva Srivastava

**Affiliations:** 1 Department of Biosciences and Bioengineering, IIT Bombay, Mumbai, India; 2 Proteomics Laboratory, National Centre for Cell Science, Ganeshkhind, Pune India; 3 Advanced Centre for Treatment, Research and Education in Cancer (ACTREC) and Tata Memorial Hospital, Tata Memorial Centre, Kharghar, Navi Mumbai, Mumbai India

## Abstract

Glioblastoma multiforme (GBM), the most malignant of all gliomas is characterized by a high degree of heterogeneity and poor response to treatment. The sub-ventricular zone (SVZ) is the major site of neurogenesis in the brain and is rich in neural stem cells. Based on the proximity of the GBM tumors to the SVZ, the tumors can be further classified into SVZ+ and SVZ−. The tumors located in close contact with the SVZ are classified as SVZ+, while the tumors located distantly from the SVZ are classified as SVZ−. To gain an insight into the increased aggressiveness of SVZ+ over SVZ− tumors, we have used proteomics techniques like 2D-DIGE and LC-MS/MS to investigate any possible proteomic differences between the two subtypes. Serum proteomic analysis revealed significant alterations of various acute phase proteins and lipid carrying proteins, while tissue proteomic analysis revealed significant alterations in cytoskeletal, lipid binding, chaperone and cell cycle regulating proteins, which are already known to be associated with disease pathobiology. These findings provide cues to molecular basis behind increased aggressiveness of SVZ+ GBM tumors over SVZ− GBM tumors and plausible therapeutic targets to improve treatment modalities for these highly invasive tumors.

## Introduction

Glioblastoma Multiforme (GBM) is a malignant brain tumor, characterized by heterogeneity within the tumor and poor response to treatment^1^. It is also the most aggressive of all known gliomas with patients having a survival period lower than one year from the time of diagnosis in most cases^2^. WHO categorizes GBMs as grade IV gliomas^3^. Progenitor astrocytes that go on to form glial cells colonize the subventricular zone (SVZ), the largest germinal zone in the brain found along the lateral walls of the lateral ventricles^4^. This zone of the brain has been extensively studied as a site of neurogenesis^5^ and is responsible for the development of neurons in an adult. The aggressive nature of GBM tumors makes their treatment almost impossible thereby making them the most dangerous of all known gliomas. Surgical resection of the tumor tissues followed by radiotherapy (RT) and biopsy followed by radiotherapy (RT) have been reported to prolong the survival period in the patients with GBMs^6^.

Despite all the advances in proteomics there still exists a huge void in our understanding of GBMs and the proteins with possible role in survival of the patients. A proteome based clustering of gliomas was correlated to survival in patients^7^. Haskin *et al*. compared the tissue proteome of GBM tumors (tumors in close proximity to the SVZ region) with the control tissues and identified various protein signatures responsible for migratory potential and tumor growth in GBM tumors^8^. Till date there has been no report of any study involving two different sub-groups of GBMs based on SVZ involvement. Tumor proximity to the subventricular zone has been recently reported to play a significant role in the survival of GBM patients, where patients with tumors in SVZ contact had an overall survival period less than those with tumors distantly located to SVZ^9^. However, there has been no proteomic study to support the findings at a molecular level. In the current study, we have performed a comprehensive serum and tissue proteome analysis of two sub-populations of GBMs to explore if SVZ involvement had any direct influence on tumor aggressiveness.

## Results

### Serum proteomic alterations in SVZ+ patients with respect to SVZ− patients

**Figure 1:**
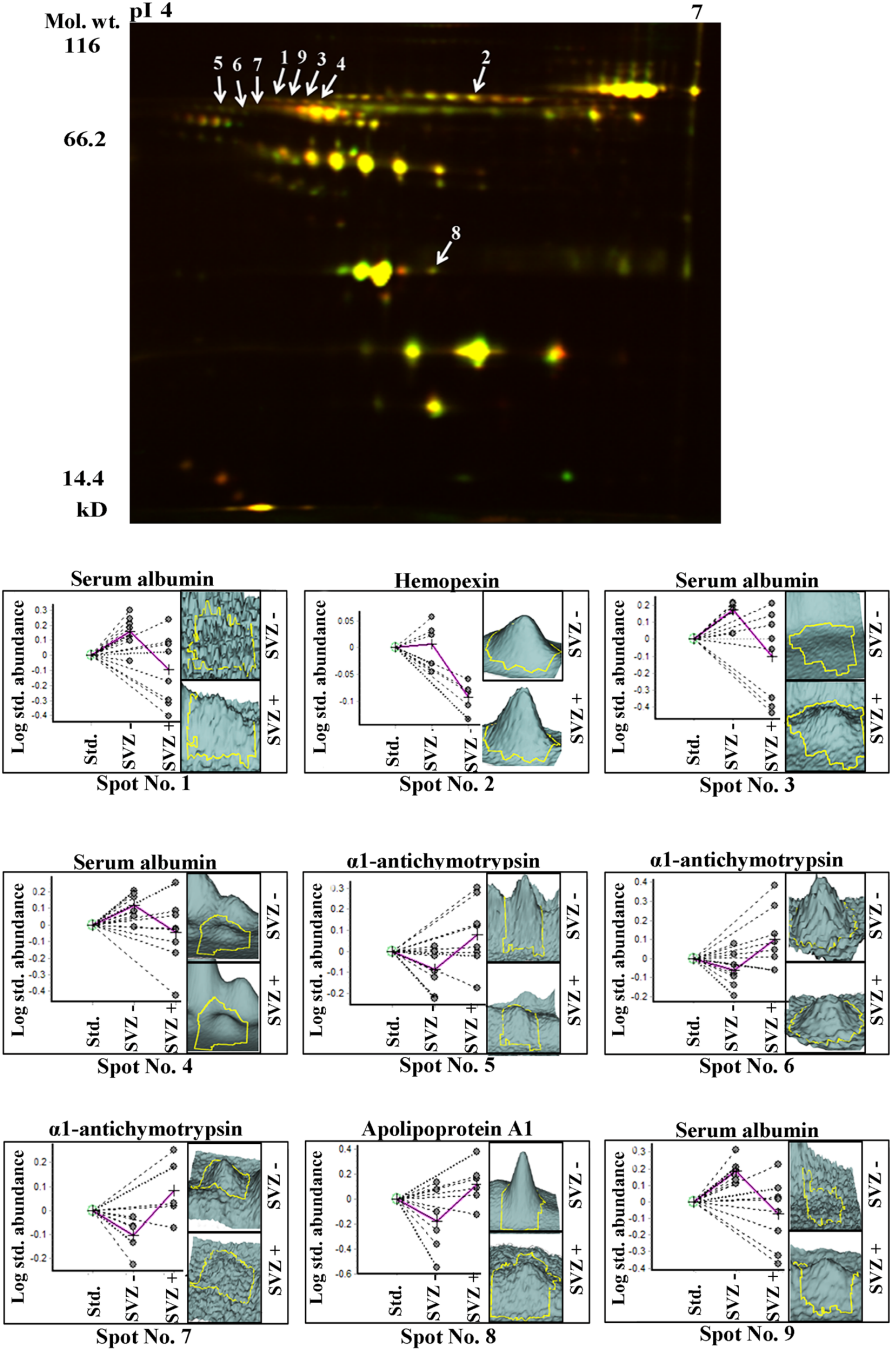
Representative overlapped 2D-DIGE gel image of SVZ+ and SVZ- GBM patient’s serum proteome and 3D-views of significantly altered proteins. Extracted serum proteome from SVZ+ (n = 8) and SVZ- (n = 8) GBM patients were labeled with respective CyDyes and separated on IPG strips of pH 4–7; 18 cm followed by separation on 12.5% SDS polyacrylamide gels. 2D-DIGE analysis was performed using DeCyder 2D software version 7.0 and the identities of significantly altered protein spots were established using MALDI-TOF/TOF analysis (Green colour indicates Cy3 labeled protein and red colour indicates Cy5 labeled protein).

The 2D-DIGE gel for serum of SVZ+ and SVZ− GBM patients yielded ~700 protein spots. Of these ~700 spots, 10 protein spots were found to be significantly altered with a *p*-value ≤ 0.05. Out of the 10 protein spots, six protein spots were found to be up-regulated and four were down-regulated in the serum of SVZ+ GBM patients. Protein identity was revealed for nine out of the 10 significantly altered protein spots using MALDI-TOF/TOF. It was found that 10 protein spots corresponded to four different proteins because of the presence of isoforms and multiple sub-units in the proteins, thereby resulting in same protein hits for multiple spots. Out of the four proteins identified, two were up-regulated and two were down-regulated in the SVZ+ GBM serum samples (Table 1). Figure 1 represents the 2D-DIGE gel image and 3D-views for the significantly altered proteins in serum.

**Table 1:** List of the differentially expressed and statistically significant protein spots from SVZ +/− GBM patients serum and tissue samples identified using 2D-DIGE method.

**(A) Significantly altered proteins in serum of SVZ +/− GBM patients identified using 2D-DIGE**							
**Spot No**.	**Name of the protein**	**Uniprot accession number**	**Mol. Wt (kDa)**	***p*** **value (** ***t-*** **test)**	**Fold change (SVZ+/SVZ−)**	**Protein score**	**No. of matched peptides**
1	Serum albumin	P02768	71.31	0.014	1.63	191	21
2	Hemopexin*	P02790	52.38	0.0065	1.21	883	24
3	Serum albumin	P02768	71.31	0.011	1.64	137	20
4	Serum albumin	P02768	71.31	0.049	1.36	293	26
5	α1-antichymotrypsin	P01011	47.79	0.025	−1.52	220	19
6	α1-antichymotrypsin	P01011	47.79	0.025	−1.51	244	18
7	α1-antichymotrypsin^$^	P01011	47.79	0.026	−1.58	284	20
8	Apolipoprotein A1*	P02647	30.75	0.044	−1.82	308	25
9	Serum albumin	P02768	71.31	0.0039	1.68	181	18
**(B) Significantly altered proteins in tissue of SVZ +/− GBM patients identified using 2D-DIGE**							
**Spot No**.	**Name of the protein**	**UniProt accession number**	**Mol. Wt (kDa)**	**p value (t-test)**	**Fold change (SVZ+/SVZ−)**	**Sequest score**	**Unique peptides**
1	Autoantigen La (Lupus La)	P05455	46.8	0.024	−1.87	146.41	23
2	Proliferation associated 2G4 homologue	Q9UQ80	40.9	0.049	−2.33	176.5	23
3	Retinol binding protein 1	P09455	15.8	0.011	−2.89	35.14	7
4	Rab GDP dissociation protein alpha	P31150	50.6	0.044	2.1	382.23	32
	Vimentin	P08670	53.6	0.044	2.1	287.8	41
5	Vimentin	P08670	53.6	0.049	2.4	224.25	36
	Rab GDP Dissociation Protein Alpha	P31150	50.6	0.049	2.4	214.65	27

Note: The proteins with *mark are significant in only 18 out of 24 gels and the proteins with ^**$**^mark are significant in 15 out of 24 gels. Remaining proteins are significant in all 24 out of 24 gels.

**Figure 2:**
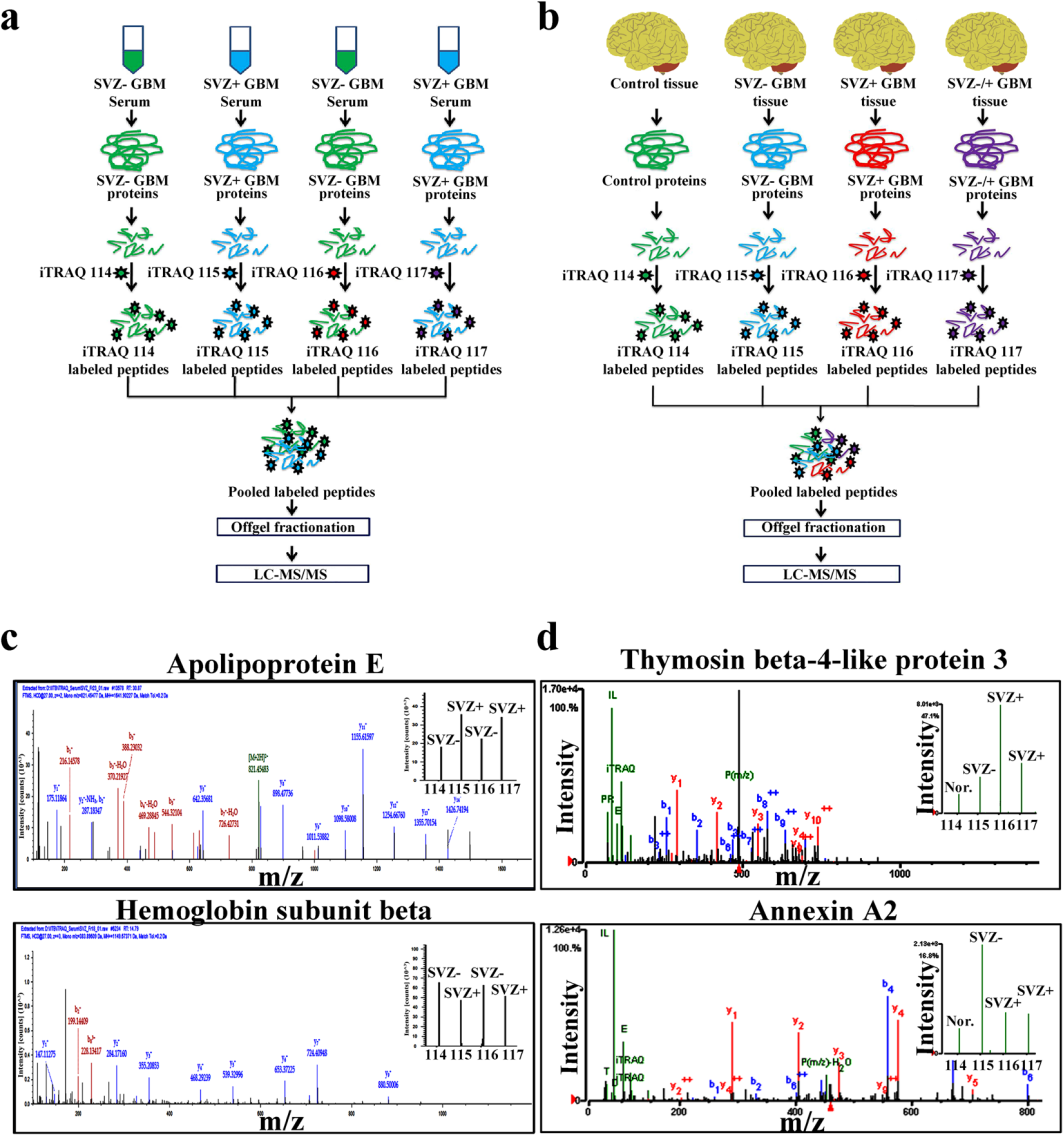
Quantitative serum and tissue proteomic analysis of SVZ+ and SVZ- GBMs. Schematic of iTRAQ based proteomic analysis of (**a**) serum and (**b**) tissue specimens for the identification of quantitative proteomic alterations. (**c**) Serum proteomic analysis of SVZ+ (n = 12) and SVZ- (n = 12) GBMs was performed using iTRAQ method. Representative mass spectra for one of the highly up-regulated (apolipoprotein E) and down-regulated (hemoglobin subunit beta) proteins in SVZ+ GBMs (w.r.t SVZ− GBMs). (**d**) Tissue proteomic analysis of SVZ+ (n = 6) and SVZ- (n = 6) GBMs was performed using iTRAQ approach. Representative mass spectra of two differentially expressed proteins, thymosin beta-4-like protein 3 and annexin A2 in SVZ+ GBM tumors (w.r.t SVZ− GBMs). Relative abundance of thymosin beta-4-like protein 3 and annexin A2 protein are shown in the inset as the intensities of iTRAQ reporter ions.

Serum proteomic analysis of SVZ+ and SVZ− GBM patients using iTRAQ method identified a total of 190 proteins, of which 148 proteins were identified with quantifiable peptides. Out of these 148 proteins, only 135 proteins showed at least ~1.1 fold change and only 75 proteins were identified with at least two peptide signatures. Of these 75 proteins, only 18 proteins showed more than 1.3 fold change (Table 2). Since the comparison involved two sub-types of GBMs, a low fold change cut-off (1.3 fold) was used to make sure that the biologically significant proteins were not missed in the discovery phase. The mass spectra of two significantly altered proteins have been provided in Fig. 2c.

**Table 2:** List of significantly altered serum and tissue proteins in SVZ +/− GBM tumor samples with respect to normal brain tissue identified using iTRAQ method (Partial list of significantly altered proteins is given in the table).

**A. Significantly altered serum proteins in SVZ+/- GBM tumors identified using iTRAQ method**					
**S. No**.	**Protein Name**	**Accession number**	**Fold Change (SVZ+/SVZ−)**	**No. of unique peptides**	
1	Vitamin D-binding protein	P02774	1.59	2	
2	Putative uncharacterized protein DKFZp686I04196	Q6N093	1.56	4	
3	cDNA, FLJ94361, highly similar to *Homo sapiens* serine (or cysteine) proteinase inhibitor	B2R9F2	1.45	5	
4	Apolipoprotein E	P02649	1.42	8	
5	cDNA FLJ60316, highly similar to Apolipoprotein-L1	B4DNT5	1.37	8	
6	Immunoglobulin J chain	C9JA05	1.33	2	
7	Apolipoprotein C-III	P02656	1.30	4	
8	cDNA FLJ56954, highly similar to Inter-alpha-trypsin inhibitor heavy chain H1	B7Z539	1.30	10	
9	Hemoglobin subunit delta	P02042	0.77	3	
10	Apolipoprotein A-IV	P06727	0.76	23	
11	Immunoblobulin light chain	Q0KKI6	0.74	5	
12	cDNA FLJ58441, highly similar to Attractin	B4DZ36	0.74	3	
13	cDNA FLJ54622, highly similar to Prothrombin	B4DDT3	0.73	4	
14	cDNA FLJ53950, highly similar to Angiotensinogen	B4E1B3	0.70	9	
15	cDNA FLJ41552 fis, clone COLON2004478, highly similar to Protein Tro alpha1 H, myeloma	Q6ZW64	0.69	4	
16	Serum amyloid A protein	Q15423	0.59	2	
17	Carbonic anhydrase 1	E5RJF6	0.58	2	
18	Hemoglobin subunit beta	P68871	0.39	2	
**B. Significantly altered tissue proteins in SVZ+/- GBM tumors identified using iTRAQ method**					
**S. No**.	**Protein Name**	**Accession number**	**Avg. Fold Change**		
			**SVZ−/Nor.**	**SVZ+/Nor.**	**SVZ+/SVZ−**
1	Alpha-1-acid glycoprotein 1	P02763	0.91	2.52	2.78
2	Band 4.1-like protein 3	Q9Y2J2	0.33	0.74	2.20
3	Thymosin beta-4-like protein 3	Q08EQ4	1.20	2.54	2.12
4	Myelin basic protein	P02686	0.30	0.63	2.09
5	Alpha-1-antitrypsin	P01009	1.41	2.71	1.93
6	Hemoglobin subunit beta	P68871	1.27	2.41	1.90
7	Haptoglobin	P00738	1.06	1.94	1.82
8	2′,3′-cyclic-nucleotide 3′-phosphodiesterase	P09543	0.32	0.57	1.79
9	Ferritin light chain	P02792	1.76	3.12	1.77
10	V-type proton ATPase catalytic subunit A	P38606	0.60	1.02	1.70
11	Hemoglobin subunit alpha	P69905	1.31	2.22	1.70
12	Vitamin D-binding protein	P02774	2.04	3.46	1.70
13	Ferritin heavy chain	P02794	0.90	1.52	1.69
14	Citrate synthase, mitochondrial	O75390	0.48	0.80	1.68
15	Astrocytic phosphoprotein PEA-15	Q15121	1.09	1.69	1.55
16	Cytochrome c oxidase subunit 5A, mitochondrial	P20674	0.33	0.51	1.54
17	Inter-alpha-trypsin inhibitor heavy chain H4	Q14624	2.00	3.06	1.53
18	Ceruloplasmin	P00450	1.32	1.96	1.48
19	Alpha-2-macroglobulin	P01023	1.04	1.54	1.48
20	Serum albumin	P02768	2.17	3.19	1.47
21	Tenascin	P24821	1.67	2.46	1.47
22	Dihydropteridine reductase	P09417	0.61	0.89	1.45
23	T-complex protein 1 subunit beta	P78371	1.55	2.24	1.44
24	Spectrin alpha chain, non-erythrocytic 1	Q13813	0.47	0.67	1.44
25	Ig alpha-1 chain C region	P01876	1.42	2.05	1.44
26	V-type proton ATPase subunit B, brain isoform	P21281	0.50	0.72	1.43
27	Microtubule-associated protein tau	P10636	0.39	0.55	1.42
28	Septin-2	Q15019	1.19	1.70	1.42
29	Ig gamma-1 chain C region	P01857	1.46	2.06	1.41
30	Aspartate aminotransferase, cytoplasmic	P17174	0.51	0.71	1.41
31	Guanine nucleotide-binding protein G(o) subunit alpha	P09471	0.37	0.52	1.40
32	Serotransferrin	P02787	1.43	2.00	1.39
33	S-formylglutathione hydrolase	P10768	0.91	1.26	1.38
34	Tryptophan–tRNA ligase, cytoplasmic	P23381	1.30	1.79	1.37
35	Microtubule-associated protein 2	P11137	0.52	0.71	1.35
36	Coactosin-like protein	Q14019	1.18	1.59	1.35
37	Synapsin-1	P17600	0.35	0.47	1.34
38	Band 4.1-like protein 2	O43491	0.74	0.99	1.34
39	Sodium/potassium-transporting ATPase subunit alpha-1	P05023	1.15	1.51	1.32
40	Ig mu chain C region	P01871	0.86	1.13	1.31
41	Glutamate dehydrogenase 1, mitochondrial	P00367	0.75	0.98	1.31
42	T-complex protein 1 subunit zeta	P40227	1.03	1.35	1.31
43	Myosin-9	P35579	1.48	1.93	1.30
44	Pyruvate kinase isozymes M1/M2	P14618	1.65	1.28	0.77
45	Alpha-crystallin B chain	P02511	1.64	1.27	0.77
46	Glial fibrillary acidic protein	P14136	1.91	1.47	0.77
47	Proteasome subunit beta type-1	P20618	1.36	1.05	0.77
48	Angiotensinogen	P01019	1.49	1.14	0.77
49	Annexin A2	P07355	2.40	1.85	0.77
50	Fructose-bisphosphate aldolase A	P04075	1.03	0.80	0.77
51	Chitinase-3-like protein 1	P36222	2.38	1.83	0.77
52	Stress-induced-phosphoprotein 1	P31948	1.12	0.86	0.77
53	Sorcin	P30626	1.68	1.29	0.77
54	Elongation factor 1-delta	P29692	1.75	1.34	0.76
55	Heterogeneous nuclear ribonucleoproteins A2/B1	P22626	1.47	1.13	0.76
56	Phosphoglucomutase-1	P36871	1.62	1.23	0.76
57	RNA-binding motif protein, X chromosome	P38159	2.08	1.57	0.75
58	Far upstream element-binding protein 2	Q92945	2.98	2.23	0.75
59	Neural cell adhesion molecule 1	P13591	0.52	0.38	0.74
60	Profilin-1	P07737	2.19	1.62	0.74
61	Ubiquitin-conjugating enzyme E2 N	P61088	0.84	0.62	0.74
62	Transthyretin	P02766	1.99	1.44	0.72
63	ES1 protein homolog, mitochondrial	P30042	1.15	0.83	0.72
64	Elongation factor 1-gamma	P26641	2.25	1.62	0.72
65	Malate dehydrogenase, mitochondrial	P40926	0.87	0.62	0.72
66	Gamma-synuclein	O76070	0.39	0.28	0.71
67	Actin-related protein 2	P61160	2.01	1.42	0.71
68	6-phosphogluconolactonase	O95336	2.21	1.56	0.70
69	Keratin, type I cytoskeletal 10	P13645	1.20	0.83	0.70
70	Heat shock protein beta-1	P04792	2.02	1.40	0.69
71	Peroxiredoxin-6	P30041	1.49	1.02	0.69
72	Trifunctional enzyme subunit beta, mitochondrial	P55084	1.43	0.97	0.68
73	Voltage-dependent anion-selective channel protein 2	P45880	0.39	0.27	0.68
74	Adenosylhomocysteinase	P23526	2.04	1.39	0.68
75	Proteasome activator complex subunit 2	Q9UL46	4.95	3.36	0.68
76	T-complex protein 1 subunit gamma	P49368	1.32	0.89	0.68
77	ADP/ATP translocase 3	P12236	0.78	0.52	0.67
78	Alpha-actinin-1	P12814	2.65	1.77	0.67
79	Retinal dehydrogenase 1	P00352	1.30	0.84	0.65
80	Cytosol aminopeptidase	P28838	2.26	1.45	0.64
81	Peptidyl-prolyl cis-trans isomerase B	P23284	2.48	1.57	0.63
82	Nucleoside diphosphate kinase B	P22392	1.68	1.06	0.63
83	Peptidyl-prolyl cis-trans isomerase A	P62937	1.04	0.64	0.62
84	Transgelin	Q01995	2.31	1.38	0.60
85	Phosphoglycerate kinase 1	P00558	1.63	0.98	0.60
86	Myristoylated alanine-rich C-kinase substrate	P29966	1.27	0.76	0.59
87	Annexin A1	P04083	2.92	1.73	0.59
88	Tenascin-R	Q92752	0.98	0.58	0.59
89	X-ray repair cross-complementing protein 6	P12956	2.28	1.29	0.56
90	Fibrinogen beta chain	P02675	2.79	1.33	0.48
91	Polypyrimidine tract-binding protein 1	P26599	1.91	0.90	0.47
92	Histone H3.1t	Q16695	3.65	1.62	0.44
93	Histone H2B type 1-M	Q99879	2.36	1.04	0.44
94	Protein dpy-30 homolog	Q9C005	2.31	1.01	0.44
95	Protein S100-A9	P06702	3.92	1.65	0.42
96	X-ray repair cross-complementing protein 5	P13010	2.01	0.83	0.41

### Tissue proteomic alterations in the sub-groups of GBM patients

**Figure 3:**
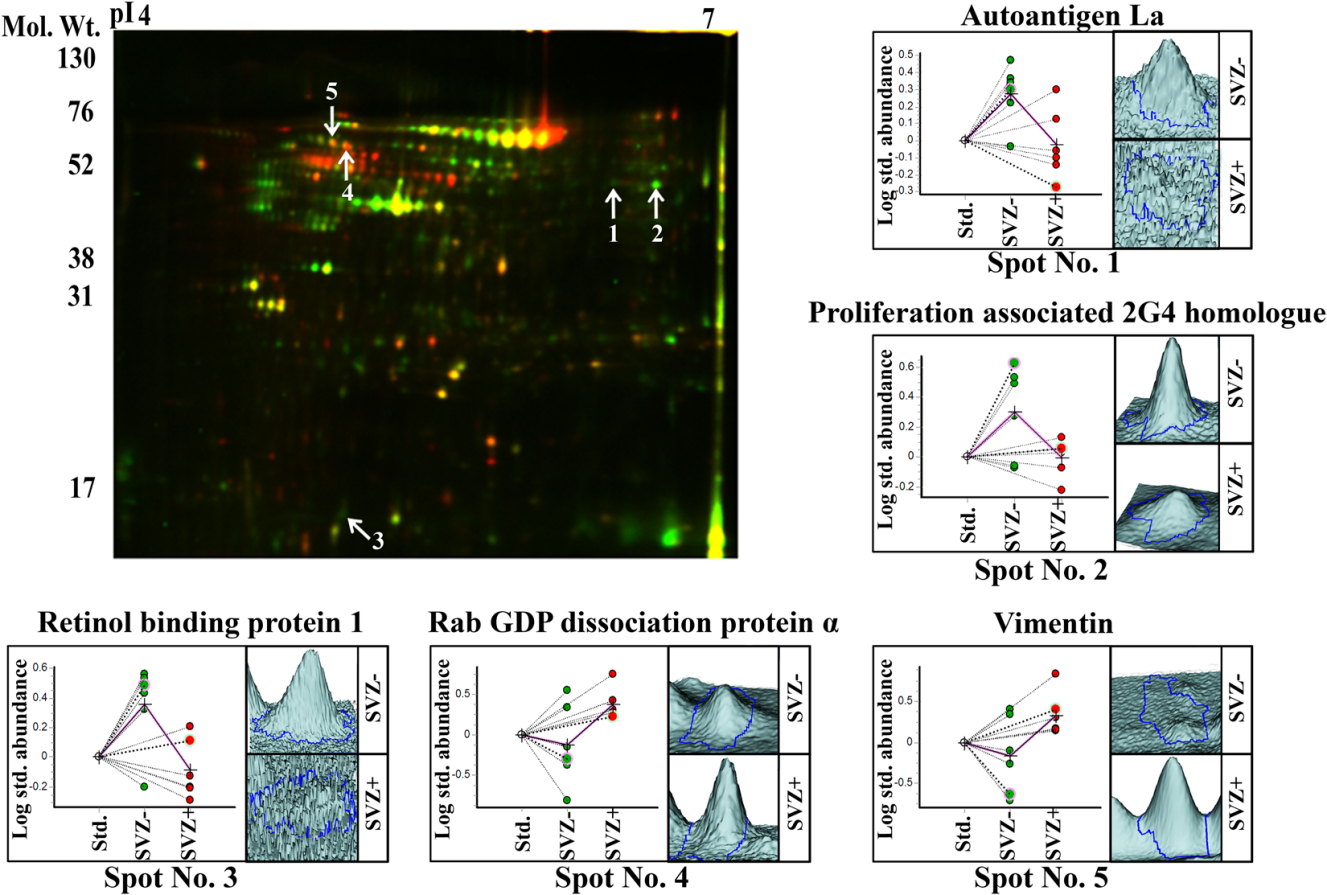
Tissue proteomic analysis of SVZ+ (n = 6) and SVZ- (n = 6) GBMs using 2D-DIGE method. Cy3 and Cy5 overlapped 2D-DIGE gel image of SVZ+ and SVZ- GBM brain tumor tissue proteome and the 3D-views for the significantly altered proteins. The tissue proteins were separated based on their pI using IPG strips of pH 4–7; 18 cm length, followed by separation based on molecular weights using 12.5% SDS-polyacrylamide gels and image analysis was performed using DeCyder 2D software. Significantly altered protein spots were subjected to MALDI-TOF/TOF analysis to establish their identity. Dot plots and 3D-views represent the relative abundance of significantly altered proteins in the SVZ+ and SVZ- GBMs (Green colour indicates Cy3 labeled protein and red colour indicates Cy5 labeled protein).

Tissue proteome of the SVZ+ and SVZ− GBM patients was studied using 2D-DIGE and iTRAQ methods. 2D-DIGE analysis was performed for the identification of tissue proteomic alterations in SVZ+ and SVZ− GBM tumor tissue samples (n = 6 each). Analysis of the 2D-DIGE gels of the GBM tumor tissue proteome was performed using DeCyder software. Approximately, 1650 protein spots were identified on each 2D-DIGE gel. After matching the gels, only five protein spots were found to be statistically significant having a *p*-value ≤ 0.05. Of the five significantly altered protein spots, two were up-regulated and three were down-regulated in SVZ+ GBM patients. These five protein spots were subjected to mass spectrometric analysis to establish their identity (Table 1; Fig. 3).

Tissue proteome of normal brain tissue was compared with the tissue proteome of SVZ+ and SVZ− GBMs using iTRAQ method. In the six comparisons possible, proteins with two peptide signatures following similar trends in more than three comparisons and with an average fold change of 1.3 were considered significant. In the first comparison involving SVZ− GBM tissue proteome and normal brain tissue proteome, 165 proteins were found to be differentially regulated with 112 proteins being up-regulated and 53 proteins down-regulated in the SVZ− GBM tumors. The comparison of SVZ+ GBM tissue proteome with the normal brain tissue proteome, yielded 164 proteins with altered levels of expression (107 proteins being up-regulated while 57 proteins being down-regulated in SVZ+ tumors). The comparison between proteomes of SVZ+ GBM tumors and SVZ− GBM tumors indicated 96 significantly differentially regulated proteins. Of these, 43 proteins were found to be up-regulated while 53 proteins were found to be down-regulated in SVZ+ tumors in comparison with SVZ− tumors. Representative mass spectra for thymosin beta-4-like protein 3 and annexin A2 have been provided in Fig. 2d. Comparison of the results arising from serum and tissue proteomic analyses using 2D-DIGE and iTRAQ methods revealed a few common proteins as mentioned in Supplementary Table s4.

Tissue proteomic alterations in SVZ+ and SVZ- GBM tumors were further compared with the mRNA expression data of long-term survivors and short-term survivors of GBM patients from TCGA. We identified 42 common proteins/genes from our proteomics data and TCGA mRNA data. Of the 42 common proteins/genes only 18 proteins/genes showed similar expression trends some of which included adenosylhomocysteinase, protein disulfide-isomerase, endoplasmin and alpha-2-macroglobulin (Supplementary Table s5). Though the TCGA survival data does not mention about SVZ involvement with tumors, we believe that our analysis would be useful in understanding the factors affecting survival in GBM patients.

Bioinformatics analysis was performed for significantly altered proteins identified from the 3 comparisons (SVZ− vs Nor., SVZ+ vs Nor., SVZ+ vs SVZ- tissue proteome analysis) using DAVID version 6.7. Most of the pathways affected in both SVZ+ and SVZ- GBMs were found to be common and associated with sugar metabolism and blood coagulation cascades.

The data was further subjected to partial least square discriminant analysis and resulted in separation of SVZ+ GBMs from SVZ- GBMs on the 3D-score plot (Supplementary Fig. s2).

### Validation of few significantly altered serum and tissue proteins

**Figure 4:**
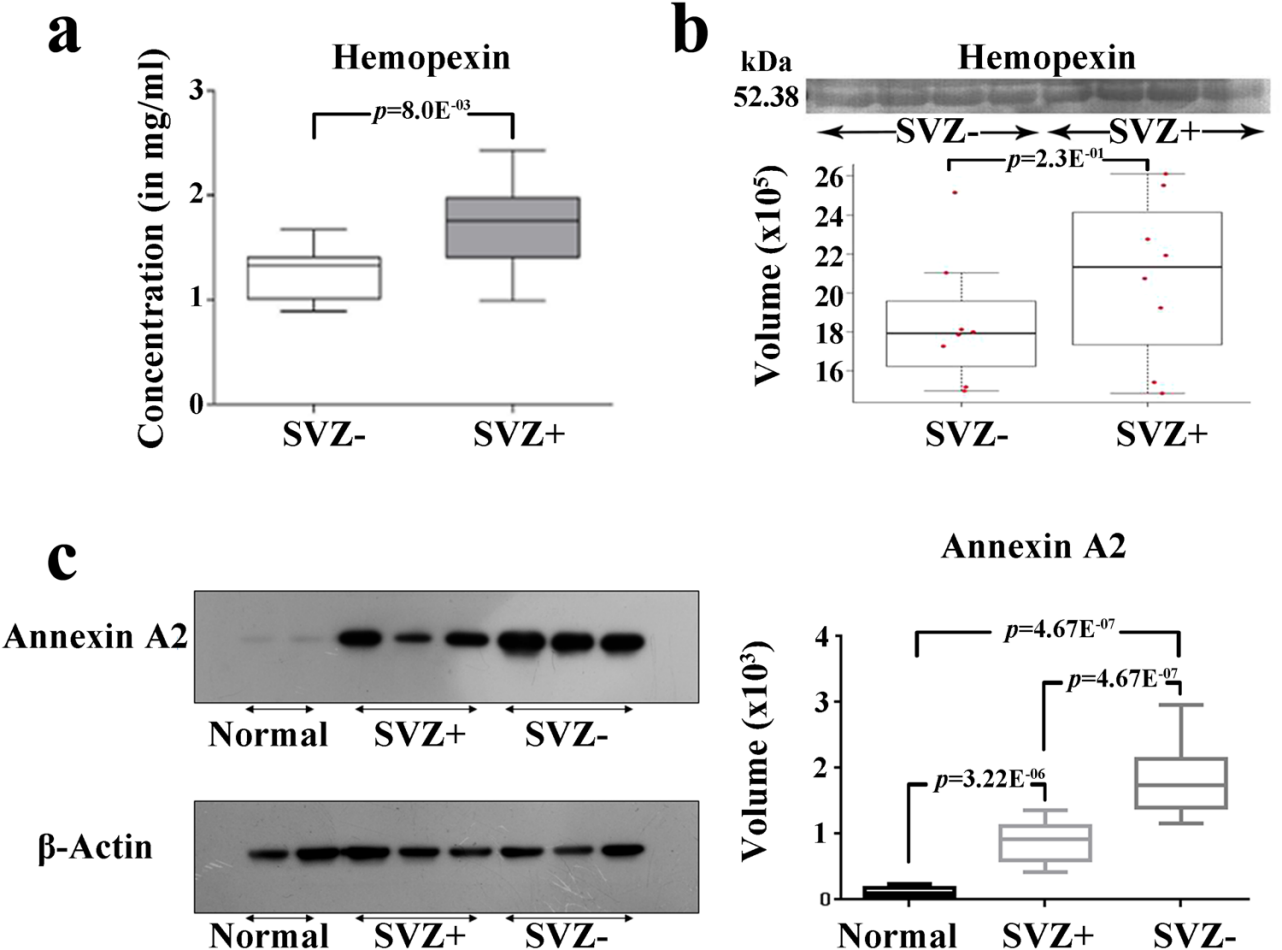
Validation of significantly altered serum (hemopexin-HPX) and tissue (annexin A2-ANXA2) proteins in SVZ+ and SVZ- GBMs. (**a**) Box plots representing the serum levels of HPX in SVZ− (n = 10) and SVZ+ (n = 10) GBMs estimated using ELISA and (**b**) western blotting results representing the serum levels of HPX in SVZ− (n = 8) and SVZ+ (n = 8) GBMs. (**c**) Validation of ANXA2 levels in normal brain tissue (n = 2), SVZ+ (n = 3) and SVZ- (n = 3) GBM tumors using western blotting method.

Hemopexin, one of the significantly up-regulated proteins in the serum samples of SVZ+ patients was validated using ELISA and western blotting methods. Serum levels of hemopexin from SVZ+ (n = 10) and SVZ− (n = 10) GBM patients were determined using competitive sandwich ELISA method. The serum levels of hemopexin in SVZ+ GBM patients (1.72 mg/ml) were found to be marginally higher than the SVZ− GBM patients (n = 1.25 mg/ml) and have been represented in graphical form in Fig. 4a.

Serum western blotting analysis for hemopexin indicated a marginal increase in the levels of the protein in SVZ+ individuals in comparison to SVZ− individuals (Fig. 4b).

Tissue western blotting analysis for annexin A2 revealed two fold up-regulation of the protein in SVZ− GBM patients as compared to SVZ+ GBM patients (Fig. 4c).

## Discussion

The current study aimed at offering an insight into the proteomic differences in serum and tissues from the two subgroups of GBMs and was based on available literature suggesting a possible role of SVZ in survival of GBM patients^9^. The proteomics study performed by Haskins *et al*. involved comparison of SVZ+ GBM tumor proteome with control samples^8^. However, protein expression analysis of both SVZ+ and SVZ− GBM tumors is likely to reveal protein signatures specific to SVZ+ GBMs. Hence, we have performed proteomic analysis of serum and tissues from SVZ+ and SVZ- GBMs in an attempt to identify protein markers responsible for aggressive nature of these tumors.

Proteomics analysis using 2D-DIGE on serum from the GBM patients revealed differential expression of four proteins-hemopexin (HPX), apolipoprotein A1 (APOA1), alpha-1-antichymotrypsin (SERPINA3)and serum albumin (ALB). A significant change in levels of acute phase proteins, HPX and SERPINA3 was observed. Besides maintaining homeostasis, hemopexin protects cells from heme mediated oxidation^10^ and plays an important role in differentiation of oligodendrocytes and myelin sheath formation^11^. SERPINA3, a serine protease inhibitor, is known to protect tissues from action of proteases^12^. Two major events in the process of metastasis include release of the tumor cells from the site of tumor by the action of proteases on the extracellular matrix and adhesion of the migrated tumor cells to the tissues in a new location with the help of cell surface molecules. Mass spectrometric analysis revealed a decrease in serum SERPINA3 levels in SVZ+ patients with respect to SVZ− patients, though the difference was negligible. The reduced levels of SERPINA3 in serum of SVZ+ GBM patients could be one of the plausible explanations for the increased aggressiveness of SVZ+ tumors over SVZ− tumors.

Levels of APOA1, a major component of HDL involved in cholesterol transport^13^, have been reported to increase in serum of patients with pancreatic cancer, gastric cancer, ovarian cancer and glioblastomas^14–16^. Survival studies in mouse models with ovarian cancer revealed higher levels of APOA1 to play a role in increased survival^13^. Serum APOA1 levels in our study were found to be down-regulated in SVZ+ GBMs. Apolipoprotein E (APOE) forms a major component of LDL carrying cholesterol from liver to extra hepatic tissues and activates cell proliferation and anti-apoptotic cascades upon binding to LDL receptors thereby helping the cells in proliferation and evading apoptosis. It has been reported to be up-regulated in breast, colon, stomach and prostate cancers^17^. Increased levels of APOE in serum of SVZ+ GBMs possibly indicates its role in membrane synthesis and evading apoptosis.

Vitamin-D has been reported to inhibit tumor angiogenesis, increase cell adhesion and induce cellular apoptosis^18, 19^. Free vitamin-D is known to cross the blood brain barrier freely^20^. Vitamin-D binding protein binds to free vitamin-D in the serum and transports it to the target sites. We observed decreased levels of vitamin-D binding protein in SVZ− GBM serum which supports our hypothesis that the decreased protein levels possibly inhibits tumor growth by increasing availability of free vitamin-D.

iTRAQ based quantitative mass spectrometric analysis of GBM tissue (SVZ+/-) proteome revealed significant changes in expression of proteins in many important metabolic pathways. Pathways involved in carbohydrate metabolism (glycolysis/gluconeogenesis and pentose phosphate pathway), amino acid metabolism (cys and met metabolism), complement and coagulation cascades, ECM-receptor interaction and neurodegenerative diseases (Parkinson’s, Alzheimer’s and Huntington’s disease) were found to be affected in SVZ− and SVZ+ GBMs (Refer Supplementary Tables s6, s7 and s8). Mitochondrial proteins involved in oxidative phosphorylation were down-regulated in both SVZ+ and SVZ- GBMs.

Cancer cells require a higher flux of glucose into glycolysis to meet the energy needs of rapidly proliferating tumor cells and to provide pentose phosphate pathway (PPP) intermediates for the synthesis of nucleic acid precursors^21^. Also, many key enzymes involved in glycolysis and PPP show increased activity in cancerous cells^22^. However, our study indicated down-regulation of a few glycolytic enzymes. It is possible that post translational modifications like phosphorylation/dephosphorylation of these enzymes control the flux of glucose into the glycolytic pathway. Except aldolase-C, most pentose phosphate pathway proteins identified in our study were up-regulated in both SVZ+ and SVZ- GBM tumors.

Proteins involved in complement pathway and blood coagulation cascades were found to be altered in both SVZ+ and SVZ- GBMs. Coagulation cascade related genes were found to be over expressed in epithelial cancer, ovarian cancer^23^ and astrocytomas^24^. Activation of the blood coagulation system was associated with poor prognosis^25^, while increased tissue factor (activator of blood coagulation system) correlated with the decreased survival of breast cancer patients^26^. Many proteins associated with blood coagulation system were found to be up-regulated in SVZ+ GBMs with respect to SVZ− GBMs in our study. Protease inhibitors like alpha-2-macroglobulin (A2M), alpha-1-antitrypsin (SERPINA1) and antithrombin-III (SERPINC1) associated with blood coagulation system, were found to be up-regulated in SVZ+ GBM tumors as compared to SVZ− GBM tumors. A2M binds to a number of proteases at their active sites using its bait region. On proteolysis, A2M changes its conformation rendering the protease inactive^27^. A2M was found to be expressed in many different cancers like human gliomas, melanomas, colon cancer etc.^28–30^ indicating its role in tumorigenicity^30^. SERPINA1, another important protease inhibitor with affinity for inhibition of neutrophil elastase^31^, is known to play an important role in modulating immunity, inflammation, apoptosis etc.^32^ and has been reported to protect brain tumor cells from the action of other proteases^33^. Fibrin/fibrinogen, a blood coagulation factor was found to be deposited in various cancers like brain tumors, prostate cancers, mesothelioma, colon cancer and lymphoma^34–38^. Fibrinogen plays critical role in angiogenesis, tumor cell growth, proliferation and development by sequestering the growth factors like fibroblast growth factor-2 and vascular endothelial growth factor^39, 40^. Tumor cells utilize sequestered growth factors by degrading the fibrinogen^41^.

Fibrin increases the metastatic capacity of tumor by virtue of its ability to bind to various cell surface molecules like integrins and non integrin receptors, thereby helping the circulating tumor cells to adhere to the cells at new location. Increased levels of fibrinogen induce plasmin, a protease and increase the invasive and metastatic nature of tumors by degrading the extracellular matrix^42^. Fibrin also protects the tumor cells from host mediated inflammatory response against the tumor cells^43, 44^.

Extracellular matrix (ECM)-receptor interaction pathways which play a role in tumor cell migration and adhesion were found to be altered in GBMs in the current study. Extracellular matrix proteins like collagen and fibronectin (FN1) were found to be up-regulated in SVZ+ GBM tumors. Collagen VI is an important extracellular matrix protein and plays a role in providing structural support to the cells, cell signaling and in increasing the availability of various growth factors^45^. Cattaruzza *et al*. studied the role of collagen VI and NG2/chondroitin sulphate proteoglycan 4 (CSPG4) molecular interactions in progression of soft tissue sarcomas. Patients suffering from soft tissue sarcomas with over-expressed Collagen VI and CSPG4 together were found to have the worst disease free survival rates^46^. Interaction of Collagen VI with NG2/CSPG4 resulted in increased cell motility in human glioma cell lines^47^. Col VI was found to protect the cells from apoptosis by activating Akt/PI3K pathway^48^ and has been reported to be over expressed in breast cancer, ovarian cancer and gliomas^45, 49, 50^. Fibronectin, a glycoprotein with cell adhesive regions on it, plays a major role in the tumor cell migration and invasion through its interaction with partner proteins called integrins^51^. Increased levels of fibronectin have been reported to cause an increase in migration rate of glial tumors^52^, in addition to increasing the invasive nature of the tumors by up-regulating a protein tyrosine kinase tie2, which activates PI3-kinase and PAK^53^. We found increased levels of Collagen alpha-3 (VI) chain and fibronectin in SVZ+ GBM tumors (with respect to SVZ− GBM tumors), providing a plausible explanation for their increased invasiveness over SVZ− GBM tumors. Tenascin-C, an extracellular protein majorly expressed during embryonic development is known to disappear in adults but reappears at the site of wound healing in cases of brain cancer and colorectal carcinoma^54–56^. It interferes with syndecan-4 binding to fibronectin and enhances proliferation of glioma and breast cancer cells^57^. Increase in levels of tenascin were correlated with increase in grade of tumors and invading tumor cells besides tumor cell proliferation and angiogenesis in gliomas^58^. In the current study, levels of tenascin-C were found to be increased in both SVZ+ and SVZ− tumors as compared to peritumoral tissue, with the levels of tenascin-C in SVZ+ tumors being higher than SVZ− tumors.

Some cytoskeleton associated proteins like thymosin beta 4 (TMSB4X) and brain acid soluble protein 1 (BASP1) were found to be significantly altered in SVZ+ GBMs, and showed good correlation with the aggressive nature of SVZ+ tumors. Thymosin beta-4, an actin binding protein plays a major role in actin polymerization and maintains dynamic equilibrium between G-actin and F-actin. It is also involved in the proliferation, migration, survival and aggressiveness of colorectal cancer and pancreatic cancer via activation of Akt and JNK signaling pathways, respectively^59, 60^. Expression of thymosin beta-4 was found to have a negative correlation with survival period of patients with non-small cell lung cancer (NSCLC), where the protein expression was associated with increased metastasis and poor prognosis^61^. We observed an up-regulation of thymosin beta-4 like protein 3 in SVZ+ GBM patients as compared to SVZ− GBM in the current study. BASP1 is involved in organization of cytoskeletal elements and determines the morphology of plasma membrane^62^. Hartl *et al*. showed a decrease in expression levels of *BASP1* in cells transformed with the Myc, whereas the ectopic expression of *BASP1* in the cells transfected with v-*myc* oncogene showed resistance to v-*myc* induced transformation^63^. *BASP1* over expression was shown to result in death of cells by apoptosis, whereas decreased expression of *BASP1* was found to increase cell survival^64^. In the current study, both SVZ+ and SVZ− GBM tumors showed reduced levels of BASP1, which possibly helps the tumor cells in evading apoptosis.

The proteins identified in the current study provide a molecular evidence for the aggressive nature of SVZ+ GBM tumors over SVZ− GBM tumors. However, these proteins need to be further explored in biological models i.e., glioma cell lines and animal models to authenticate their role in increased aggressiveness of SVZ+ tumors.

## Materials and Methods

### Patient selection and samples collection

GBM serum and tissue samples were collected from Tata Memorial Centre’s, Advanced Centre for Treatment, Research and Education (TMC-ACTREC), Mumbai, India. The samples were categorized into two groups based on sub-ventricular zone involvement and median age of the patients. A total of 21 tissue samples (nine SVZ−, eight SVZ+ and four peritumoral samples) and 33 serum samples (16 SVZ− and 17 SVZ+) were used in the current proteomics study. The study was carried out after approval of the institutional ethics committee of ACTREC (IEC-80) and IIT Bombay. Pre-informed consent of all the subjects enrolled for this study were taken before sample collection. All experiments were performed in accordance with guidelines and regulations laid down by institutional ethics committees of TMH and IIT Bombay. Wherever possible, peritumoral tissue samples from presumed uninvolved brain included in the resection specimen (with confirmed histology) were used as normal control samples for the proteomics study.

### Serum and brain tissue samples processing

Protein extraction from serum was performed using TCA–Acetone precipitation method^65^, whereas the brain tissue proteins were extracted using trizol method^66^. The pre-processing steps for the serum and tissue protein extraction are described in detail in the supplementary materials and methods section. The extracted protein samples were quantified using 2D-Quant kit (GE Healthcare) and the protein samples were stored at −20 °C until further use (For more details refer supplementary materials and methods).

### Two dimensional difference gel electrophoresis (2D-DIGE)

The protein samples in rehydration buffer were minimal labeled with CyDyes as per the manufacturer’s instructions (GE Healthcare), pooled and loaded onto a 18 cm IPG strip of pH 4–7 (linear) followed by isoelectric focusing (IEF) for 64 kVh (for tissue proteins) and 78 kVh (for serum proteins). After IEF, the proteins were further separated on a 12.5% SDS-Polyacrylamide gels followed by scanning the gels using Typhoon FLA 9500 scanner (GE Healthcare) at different excitation and emission wavelengths. All the images were scanned at 100 µm resolution and the images obtained were further processed using DeCyder 2D software version 7.0 (GE Healthcare) (For more details refer supplementary materials and methods).

### MALDI-TOF/TOF analysis

The significantly altered protein spots were subjected to in-gel digestion^67^ followed by mass spectrometric analysis using 4800 MALDI TOF/TOF MS (AB Sciex) in reflectron mode. The mass spectrometry data was analysed using MASCOT version 2.1 search engine for identification of the protein against Swiss-Prot database (For more details refer supplementary materials and methods).

### In-solution digestion and iTRAQ labeling

The brain tissue and serum proteins were made LC-MS compatible by exchanging the buffer with 0.5 M TEAB using 3 kDa molecular weight cutoff filters. The protein samples were subjected to denaturation, reduction and alkylation followed by overnight trypsin digestion for 16 hr. The in-solution digested normal brain tissue proteins, SVZ− and SVZ+ GBM tumor tissue protein samples were then labeled with iTRAQ reagents 114, 115 and 116, respectively. In case of first two iTRAQ sets, iTRAQ reagent 117 was used for labeling of in-solution digested SVZ− GBM tissue proteins, whereas in the iTRAQ sets three and four, iTRAQ reagent 117 was used for labeling of in-solution digested SVZ+ GBM tissue proteins. For serum proteomic analysis, trypsin digested SVZ− serum proteins were labeled with iTRAQ reagent 114 and 116, where as the SVZ+ GBM patient’s serum proteins were labeled with iTRAQ reagent 115 and 117. The labeled samples were finally pooled and subjected to off-gel fractionation (For more details refer supplementary materials and methods).

### Offgel fractionation and LC-MS/MS

200 µg of the labeled and pooled peptide sample was subjected to Offgel fractionation on 3–10 non-linear, 24 cm high resolution IPG strips using Agilent 3100 Offgel fractionator. Minor modifications were made to the manufacturer’s protocol (Agilent Technologies). Isoelectrically focused and concentrated peptide fractions were later reconstituted in 0.1% formic acid and subjected to LC-MS/MS (For more details refer supplementary materials and methods).

### Bioinformatics analysis

The mRNA expression data for 558 GBM samples was downloaded from TCGA data portal (https://gdc-portal.nci.nih.gov/) and the patients were classified into short-term survivors (STS), median-term survivors (MTS) and long-term survivors (LTS) based on their overall survival. GBM patients with an overall survival of <1 year, 1–3 years and >3 years were classified into STS, MTS and LTS, respectively. In the current study, we compared the mRNA expression data of 217 STS with 38 LTS GBMs using BRB-Array tools^68^. Using class comparison module of BRB-Array tools, the expression levels of different genes in short-term survivors of GBMs was compared with long-term survivors of GBM patients. Further, the mRNA expression data (*p-*value ≤ 0.05) was correlated with protein expression data obtained from our quantitative mass spectrometric studies.

The data obtained from the tissue proteomic analysis of the SVZ+, SVZ− GBMs and normal brain tissue proteome was further subjected to bioinformatics analysis using the Database for Annotation, Visualization and Integrated Discovery (DAVID) version 6.7. Significantly altered proteins obtained from the iTRAQ analysis of SVZ+ GBM vs Normal, SVZ− GBM vs Normal and SVZ+ vs SVZ− GBM were subjected to DAVID analysis^69, 70^ to identify the pathways affected in the sub groups of GBM patients.

iTRAQ data for the tissue proteomic analysis of SVZ−, SVZ+ GBM tumors and normal/control tissues was subjected to PLSDA using METAGENassist, an online tool^71^. Both SVZ− and SVZ+ GBMs were segregated from each other on a 3D-score plot obtained from this multivariate analysis.

### ELISA and Western blotting

Hemopexin levels in GBM patient (both SVZ+ and SVZ−) serum samples were determined using competitive sandwich ELISA method (AssayPro ELISA kit, USA) following manufacturer’s instructions. The absorbance of the chromogenic substrate was read at two different wavelengths, i.e., 450 nm and 570 nm using SpectraMax microplate reader (Molecular devices). The absorbance values at 570 nm were substracted from the values at 450 nm and the resulting absorbance values were used for determining the serum concentrations of hemopexin from the standard curve.

Western blotting was performed on the serum samples of SVZ+ (n = 8) and SVZ− (n = 8) GBM patients to validate the serum hemopexin levels. The serum proteins were separated using SDS-PAGE followed by their transfer onto PVDF membrane. Blocking was done in 5% skimmed milk followed by incubation with primary antibody (Rabbit polyclonal IgG, 1:500 dilution) against hemopexin (Santacruz biotechnology, lot no.J2909, sc-13443) followed by incubation with HRP conjugated secondary antibody (1:2500 dilution, Goat anti-Rabbit IgG-HRP, GeNei, lot no. 032071) against the primary antibody, followed by washes with TBST buffer. A chromogenic substrate (TMB/H_2_O_2_, GeNei, lot no. 033091) was added, followed by scanning the membrane using LabScan (GEHealthcare). Densitometric analysis of the hemopexin bands was performed using ImageQuant TL software (GE Healthcare).

Western blotting was carried out on SVZ+ and SVZ− GBM tissue lysates (n = 3 for each subtype) and compared with normal brain tissue lysates (n = 2) in order to validate the levels of Annexin A2. 10 µg of the protein lysates were resolved on 8% SDS-PAGE gels following which the proteins were transferred onto a PVDF membrane. Post transfer, the membrane was blocked using 5% non-fat dried milk in 0.1% TBST (pH- 7.4) for one hour. The blots were then probed with primary antibodies for Actin, which was used as loading control (Mouse polyclonal 1:2000) and Annexin A2 (Rabbit polyclonal 1:5000) overnight at 4 °C. Following overnight incubation, the blots were washed three times in 0.1% TBST and incubated for 1 hour with secondary antibodies anti- mouse (1:8000) and anti-rabbit (1:5000) for Actin and Annexin A2, respectively. The blots were finally washed three times in 0.1% TBST and developed using chemiluminescent reagent (GE Amersham) followed by autoradiography. Densitometric analysis of the blots was performed using ImageQuant TL software (GE Healthcare).

## Electronic supplementary material

Supplementary information
