# Supplementary material for: Subventricular zone involvement in Glioblastoma – A proteomic evaluation and clinicoradiological correlation
Source: Sci Rep. 2017 May 3;7:1449. doi: 10.1038/s41598-017-01202-8 (PMC5431125; doi:10.1038/s41598-017-01202-8)
Supplement: Supplementary file 1 — Supplementary information [file 41598_2017_1202_MOESM1_ESM.doc]

**Subventricular zone involvement in Glioblastoma – A proteomic evaluation and clinicoradiological correlation**

*Kishore Gollapalli^1^, Saicharan Ghantasala^1^, Sachendra Kumar^1^, Rajneesh Srivastava^1^, Srikanth Rapole^2^, Aliasgar Moiyadi^3^, Sridhar Epari^3^and Sanjeeva Srivastava^1^*

^1^Department of Biosciences and Bioengineering, IIT Bombay, Mumbai, India

^2^Proteomics Laboratory, National Centre for Cell Science, Ganeshkhind, Pune, India

^3^Advanced Centre for Treatment, Research and Education in Cancer (ACTREC) and Tata Memorial Hospital, Tata Memorial Centre, Kharghar, Navi Mumbai, Mumbai, India

Correspondence to [sanjeeva@iitb.ac.in]

**Supplementary Figures**


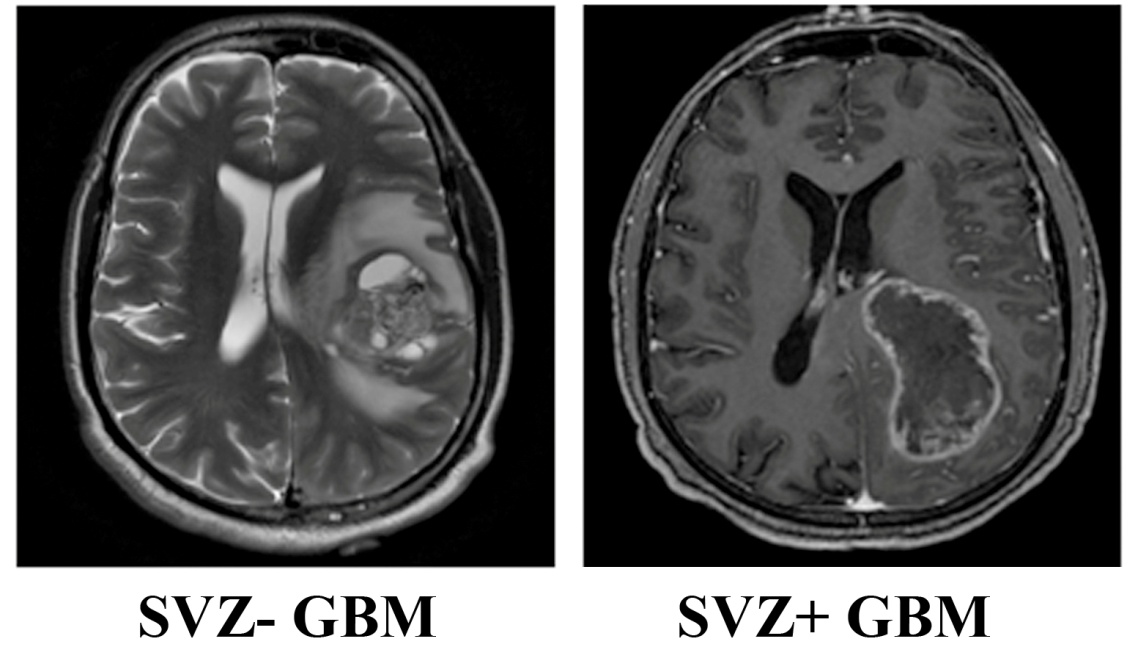


**Supplementary Fig.s1.** Representative MRI images of SVZ- and SVZ+ GBM tumors.

**
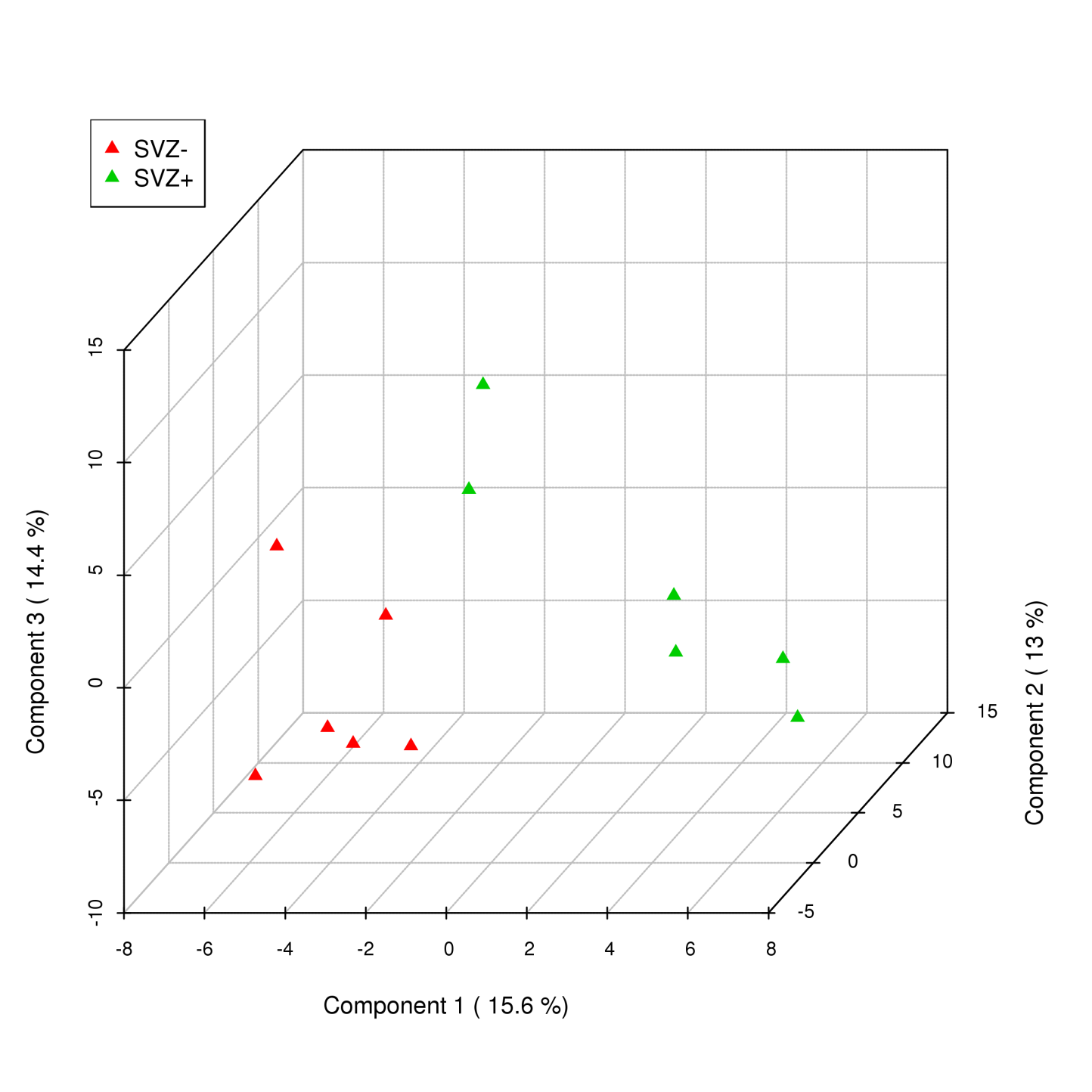
**

**Supplementary Fig.s2.** Separation of SVZ+ and SVZ- GBMs on 3D-score plot obtained from partial least square discriminant analysis (PLSDA) of iTRAQ data for SVZ+ and SVZ- GBM tumor tissue proteome. Using METAGENassist, an online tool, PLSDA was performed using the tissue proteomic analysis data from iTRAQ experiments (SVZ+ vs SVZ- GBMs) and the two subgroups of GBM were well separated on the 3D-score plot.

**Supplementary Tables:**

**Supplementary Table s**1. Clinical details of the GBM patients enrolled in the study

| Sample | Association with SVZ region | AGE | Sex | EOR | Multi-  Focality | rt_  taken | adjuvant  treatment  completed | Progression | Last follow-up  status | preop_  KPS |
| --- | --- | --- | --- | --- | --- | --- | --- | --- | --- | --- |
| GBM1 | SVZ- | 59 | M | near total | NO | yes | yes | YES | dead due to disease | 100 |
| GBM2 | SVZ+ | 74 | M | near total | NO | no | yes | YES | dead due to disease | 80 |
| GBM3 | SVZ- | 67 | M | gross total | NO | yes | yes | NO | dead due to other cause | 70 |
| GBM4 | SVZ+ | 67 | M | near total | NO | no | no | YES | dead due to disease | 30 |
| GBM5 | SVZ- | 69 | M | near total | NO | yes | yes | YES | dead due to disease | 60 |
| GBM6 | SVZ- | 57 | F | gross total | NO | no | no | YES | dead due to other cause | 70 |
| GBM7 | SVZ- | 56 | M | gross total | NO | yes | yes | YES | dead due to disease | 90 |
| GBM8 | SVZ+ | 56 | M | near total | NO | yes | no | YES | dead due to disease | 80 |
| GBM9 | SVZ- | 44 | F | gross total | NO | yes | yes | YES | dead due to disease | 70 |
| GBM10 | SVZ- | 64 | M | subtotal | NO | no | no | YES | dead due to disease | 60 |
| GBM11 | SVZ+ | 61 | M | subtotal | NO | yes | yes | YES | dead due to disease | 70 |
| GBM12 | SVZ- | 61 | M | subtotal | YES | yes | no | YES | dead due to disease | 80 |
| GBM13 | SVZ+ | 55 | F | near total | YES | no | no | YES | dead due to disease | 90 |
| GBM14 | SVZ+ | 67 | M | near total | NO | no | no | YES | dead due to disease | 90 |
| GBM15 | SVZ+ | 53 | M | subtotal | NO | no | no | YES | dead due to disease | 70 |
| GBM16 | SVZ- | 50 | M | gross total | NO | yes | yes | NO | alive with stable disease | 80 |
| GBM17 | SVZ+ | 69 | F | subtotal | NO | no | no | YES | dead due to disease | 70 |
| GBM18 | SVZ+ | 47 | M | subtotal | YES | yes | yes | NO | alive with stable disease | 90 |
| GBM19 | SVZ+ | 64 | M | gross total | YES | yes | no | YES | dead due to disease | 60 |
| GBM20 | SVZ+ | 50 | F | subtotal | NO | yes | no | YES | dead due to disease | 80 |
| GBM21 | SVZ- | 37 | M | gross total | NO | yes | no | YES | dead due to disease | 70 |
| GBM22 | SVZ- | 81 | M | gross total | NO | yes | no | YES | dead due to disease | 70 |
| GBM23 | SVZ+ | 46 | F | near total | YES | yes | yes | YES | alive with progressive disease | 90 |
| GBM24 | SVZ- | 64 | M | gross total | NO | yes | yes | NO | alive with stable disease | 60 |
| GBM25 | SVZ+ | 52 | M | near total | NO | yes | no | YES | dead due to disease | 90 |
| GBM26 | SVZ+ | 56 | M | gross total | NO | yes | unknown | YES | dead due to disease | 80 |
| GBM27 | SVZ- | 59 | M | near total | NO | yes | yes | NO | alive with stable disease | 90 |
| GBM28 | SVZ+ | 42 | F | near total | NO | unknown | unknown | unknown | lost to fu | 80 |
| GBM29 | SVZ- | 61 | M | subtotal | NO | no | no | YES | dead due to disease | 60 |
| GBM30 | SVZ+ | 56 | M | gross total | NO | yes | no | YES | dead due to disease | 70 |
| GBM31 | SVZ+ | 52 | M | gross total | NO | yes | yes | NO | alive with stable disease | 60 |
| GBM32 | SVZ- | 60 | M | gross total | NO | yes | yes | NO | alive with stable disease | 80 |
| GBM33 | SVZ- | 59 | M | near total | NO | yes | yes | NO | alive with stable disease | 80 |

**Supplementary Table s2. Serum proteomic alterations in SVZ+ and SVZ-** GBM patients identified using iTRAQ approach

| S.No. | Accession | Protein Name | Unique Peptides | Avg. Fold change (SVZ+/ SVZ-) |
| --- | --- | --- | --- | --- |
| 1 | P02774 | Vitamin D-binding protein | 2 | 1.59 |
| 2 | Q6N093 | Putative uncharacterized protein DKFZp686I04196 | 4 | 1.56 |
| 3 | B2R9F2 | cDNA, FLJ94361, highly similar to Homo sapiens serine (or cysteine) proteinase inhibitor | 5 | 1.45 |
| 4 | P02649 | Apolipoprotein E | 8 | 1.42 |
| 5 | B4DNT5 | cDNA FLJ60316, highly similar to Apolipoprotein-L1 | 8 | 1.37 |
| 6 | C9JA05 | Immunoglobulin J chain | 2 | 1.33 |
| 7 | P02656 | Apolipoprotein C-III | 4 | 1.30 |
| 8 | B7Z539 | cDNA FLJ56954, highly similar to Inter-alpha-trypsin inhibitor heavy chain H1 | 10 | 1.30 |
| 9 | P01861 | Ig gamma-4 chain C region | 2 | 1.28 |
| 10 | B4E1I8 | cDNA FLJ54228, highly similar to Leucine-rich alpha-2-glycoprotein | 5 | 1.25 |
| 11 | F8WCZ6 | Complement C1s subcomponent | 3 | 1.22 |
| 12 | A0N5G5 | Rheumatoid factor D5 light chain | 2 | 1.22 |
| 13 | P01598 | Ig kappa chain V-I region EU | 2 | 1.21 |
| 14 | B4E1B0 | cDNA FLJ54318, highly similar to Complement C1r subcomponent | 2 | 1.21 |
| 15 | P02763 | Alpha-1-acid glycoprotein 1 | 5 | 1.20 |
| 16 | P19652 | Alpha-1-acid glycoprotein 2 | 4 | 1.18 |
| 17 | P01011 | Alpha-1-antichymotrypsin | 9 | 1.14 |
| 18 | Q5T985 | Inter-alpha-trypsin inhibitor heavy chain H2 | 12 | 1.13 |
| 19 | C0JYY2 | Apolipoprotein B (Including Ag(X) antigen) | 28 | 1.13 |
| 20 | B2R5G8 | Serum amyloid A protein | 3 | 1.12 |
| 21 | Q9UL88 | Myosin-reactive immunoglobulin heavy chain variable region | 2 | 1.12 |
| 22 | Q06033 | Inter-alpha-trypsin inhibitor heavy chain H3 | 8 | 1.11 |
| 23 | P02790 | Hemopexin | 12 | 1.11 |
| 24 | P10909 | Clusterin | 10 | 1.10 |
| 25 | P01871 | Ig mu chain C region | 16 | 1.07 |
| 26 | Q6NS95 | IGL@ protein | 2 | 1.03 |
| 27 | P69905 | Hemoglobin subunit alpha | 8 | 1.03 |
| 28 | B4DPP8 | cDNA FLJ53075, highly similar to Kininogen-1 | 8 | 1.01 |
| 29 | Q6P163 | APOC2 protein | 3 | 1.00 |
| 30 | P01009 | Alpha-1-antitrypsin | 27 | 1.00 |
| 31 | P01024 | Complement C3 | 51 | 0.99 |
| 32 | Q6ZVX0 | cDNA FLJ41981 fis, clone SMINT2011888, highly similar to Protein Tro alpha1 H,myeloma | 3 | 0.99 |
| 33 | B4E1D8 | cDNA FLJ51597, highly similar to C4b-binding protein alpha chain | 3 | 0.98 |
| 34 | P00739 | Haptoglobin-related protein | 2 | 0.97 |
| 35 | Q9H804 | cDNA FLJ14022 fis, clone HEMBA1003538, weakly similar to Complement C1r component | 2 | 0.96 |
| 36 | P01008 | Antithrombin-III | 8 | 0.95 |
| 37 | Q7Z7J6 | Actin alpha 1 skeletal muscle protein | 2 | 0.94 |
| 38 | B4E1B2 | cDNA FLJ53691, highly similar to serotransferrin | 26 | 0.94 |
| 39 | P01023 | Alpha-2-macroglobulin | 47 | 0.93 |
| 40 | A4PB67 | YY1AP-related protein1 | 2 | 0.92 |
| 41 | F6KPG5 | Albumin (Fragment) | 28 | 0.91 |
| 42 | P02647 | Apolipoprotein A-I | 30 | 0.91 |
| 43 | B7Z544 | cDNA FLJ51742, highly similar to Inter-alpha-trypsin inhibitor heavy chain H4 | 12 | 0.91 |
| 44 | P43652 | Afamin | 6 | 0.91 |
| 45 | P02766 | Transthyretin | 5 | 0.91 |
| 46 | B4DX19 | cDNA FLJ57644, highly similar to Serum paraoxonase/arylesterase 1 | 3 | 0.90 |
| 47 | P04217 | Alpha-1B-glycoprotein | 9 | 0.90 |
| 48 | A8K3E4 | cDNA FLJ78367, highly similar to Homo sapiens fibrinogen | 5 | 0.89 |
| 49 | P22792 | Carboxypeptidase N subunit 2 | 2 | 0.89 |
| 50 | P81605 | Dermcidin | 3 | 0.86 |
| 51 | Q8IXC3 | PS24 | 2 | 0.86 |
| 52 | P00738 | Haptoglobin | 11 | 0.86 |
| 53 | D6RF35 | Vitamin D-binding protein | 2 | 0.85 |
| 54 | Q5VY30 | Plasma retinol-binding protein(1-182) | 3 | 0.84 |
| 55 | K7ERI9 | Truncated apolipoprotein C-I (Fragment) | 2 | 0.84 |
| 56 | B4E1Z4 | Complement factor B | 7 | 0.83 |
| 57 | P25311 | Zinc-alpha-2-glycoprotein | 11 | 0.83 |
| 58 | P05543 | Thyroxine-binding globulin | 3 | 0.82 |
| 59 | P08697 | Alpha-2-antiplasmin | 5 | 0.82 |
| 60 | P02652 | Apolipoprotein A-II | 7 | 0.80 |
| 61 | P02765 | Alpha-2-HS-glycoprotein | 4 | 0.80 |
| 62 | P05090 | Apolipoprotein D | 3 | 0.80 |
| 63 | P80108 | Phosphatidylinositol-glycan-specific phospholipase D | 6 | 0.79 |
| 64 | J3QSE5 | Phosphatidylcholine-sterol acyltransferase | 4 | 0.79 |
| 65 | P51884 | Lumican | 4 | 0.78 |
| 66 | P02042 | Hemoglobin subunit delta | 3 | 0.77 |
| 67 | P06727 | Apolipoprotein A-IV | 23 | 0.76 |
| 68 | Q0KKI6 | Immunoblobulin light chain | 5 | 0.74 |
| 69 | B4DZ36 | cDNA FLJ58441, highly similar to Attractin | 3 | 0.74 |
| 70 | B4DDT3 | cDNA FLJ54622, highly similar to Prothrombin | 4 | 0.73 |
| 71 | B4E1B3 | cDNA FLJ53950, highly similar to Angiotensinogen | 9 | 0.70 |
| 72 | Q6ZW64 | cDNA FLJ41552 fis, clone COLON2004478, highly similar to Protein Tro alpha1 H, myeloma | 4 | 0.69 |
| 73 | Q15423 | Serum amyloid A protein | 2 | 0.59 |
| 74 | E5RJF6 | Carbonic anhydrase 1 | 2 | 0.58 |
| 75 | P68871 | Hemoglobin subunit beta | 2 | 0.39 |

**Supplementary Table s**3. Brain tissue proteins which were found to be altered in SVZ+/- GBM tumors identified using iTRAQ method

| S. No. | Protein Name | Accession number | Average fold change | | |
| --- | --- | --- | --- | --- | --- |
|  |  |  | SVZ-/Nor | SVZ+/Nor. | SVZ+/SVZ- |
| 1 | Alpha-1-acid glycoprotein 1 | P02763 | 0.91 | 2.52 | 2.78 |
| 2 | Band 4.1-like protein 3 | Q9Y2J2 | 0.33 | 0.74 | 2.20 |
| 3 | Thymosin beta-4-like protein 3 | Q08EQ4 | 1.20 | 2.54 | 2.12 |
| 4 | Myelin basic protein | P02686 | 0.30 | 0.63 | 2.09 |
| 5 | Alpha-1-antitrypsin | P01009 | 1.41 | 2.71 | 1.93 |
| 6 | Hemoglobin subunit beta | P68871 | 1.27 | 2.41 | 1.90 |
| 7 | Haptoglobin | P00738 | 1.06 | 1.94 | 1.82 |
| 8 | 2',3'-cyclic-nucleotide 3'-phosphodiesterase | P09543 | 0.32 | 0.57 | 1.79 |
| 9 | Ferritin light chain | P02792 | 1.76 | 3.12 | 1.77 |
| 10 | V-type proton ATPase catalytic subunit A | P38606 | 0.60 | 1.02 | 1.70 |
| 11 | Hemoglobin subunit alpha | P69905 | 1.31 | 2.22 | 1.70 |
| 12 | Vitamin D-binding protein | P02774 | 2.04 | 3.46 | 1.70 |
| 13 | Ferritin heavy chain | P02794 | 0.90 | 1.52 | 1.69 |
| 14 | Citrate synthase, mitochondrial | O75390 | 0.48 | 0.80 | 1.68 |
| 15 | Astrocytic phosphoprotein PEA-15 | Q15121 | 1.09 | 1.69 | 1.55 |
| 16 | Cytochrome c oxidase subunit 5A, mitochondrial | P20674 | 0.33 | 0.51 | 1.54 |
| 17 | Inter-alpha-trypsin inhibitor heavy chain H4 | Q14624 | 2.00 | 3.06 | 1.53 |
| 18 | Ceruloplasmin | P00450 | 1.32 | 1.96 | 1.48 |
| 19 | Alpha-2-macroglobulin | P01023 | 1.04 | 1.54 | 1.48 |
| 20 | Serum albumin | P02768 | 2.17 | 3.19 | 1.47 |
| 21 | Tenascin | P24821 | 1.67 | 2.46 | 1.47 |
| 22 | Dihydropteridine reductase | P09417 | 0.61 | 0.89 | 1.45 |
| 23 | T-complex protein 1 subunit beta | P78371 | 1.55 | 2.24 | 1.44 |
| 24 | Spectrin alpha chain, non-erythrocytic 1 | Q13813 | 0.47 | 0.67 | 1.44 |
| 25 | Ig alpha-1 chain C region | P01876 | 1.42 | 2.05 | 1.44 |
| 26 | V-type proton ATPase subunit B, brain isoform | P21281 | 0.50 | 0.72 | 1.43 |
| 27 | Microtubule-associated protein tau | P10636 | 0.39 | 0.55 | 1.42 |
| 28 | Septin-2 | Q15019 | 1.19 | 1.70 | 1.42 |
| 29 | Ig gamma-1 chain C region | P01857 | 1.46 | 2.06 | 1.41 |
| 30 | Aspartate aminotransferase, cytoplasmic | P17174 | 0.51 | 0.71 | 1.41 |
| 31 | Guanine nucleotide-binding protein G(o) subunit alpha | P09471 | 0.37 | 0.52 | 1.40 |
| 32 | Serotransferrin | P02787 | 1.43 | 2.00 | 1.39 |
| 33 | S-formylglutathione hydrolase | P10768 | 0.91 | 1.26 | 1.38 |
| 34 | Tryptophan--tRNA ligase, cytoplasmic | P23381 | 1.30 | 1.79 | 1.37 |
| 35 | Microtubule-associated protein 2 | P11137 | 0.52 | 0.71 | 1.35 |
| 36 | Coactosin-like protein | Q14019 | 1.18 | 1.59 | 1.35 |
| 37 | Synapsin-1 | P17600 | 0.35 | 0.47 | 1.34 |
| 38 | Band 4.1-like protein 2 | O43491 | 0.74 | 0.99 | 1.34 |
| 39 | Sodium/potassium-transporting ATPase subunit alpha-1 | P05023 | 1.15 | 1.51 | 1.32 |
| 40 | Ig mu chain C region | P01871 | 0.86 | 1.13 | 1.31 |
| 41 | Glutamate dehydrogenase 1, mitochondrial | P00367 | 0.75 | 0.98 | 1.31 |
| 42 | T-complex protein 1 subunit zeta | P40227 | 1.03 | 1.35 | 1.31 |
| 43 | Myosin-9 | P35579 | 1.48 | 1.93 | 1.30 |
| 44 | Complement C4-A | P0C0L4 | 1.42 | 1.83 | 1.29 |
| 45 | Neutral alpha-glucosidase AB | Q14697 | 1.64 | 2.10 | 1.28 |
| 46 | SH3 domain-binding glutamic acid-rich-like protein | O75368 | 1.02 | 1.31 | 1.28 |
| 47 | Carbonic anhydrase 1 | P00915 | 1.26 | 1.61 | 1.28 |
| 48 | Alpha-aminoadipic semialdehyde dehydrogenase | P49419 | 1.28 | 1.64 | 1.28 |
| 49 | Ig lambda-3 chain C regions | P0CG06 | 1.46 | 1.86 | 1.28 |
| 50 | Glutathione S-transferase omega-1 | P78417 | 0.81 | 1.03 | 1.27 |
| 51 | Calreticulin | P27797 | 1.27 | 1.61 | 1.26 |
| 52 | Moesin | P26038 | 1.19 | 1.49 | 1.25 |
| 53 | Microtubule-associated proteins 1A/1B light chain 3B | Q9GZQ8 | 0.50 | 0.62 | 1.24 |
| 54 | Excitatory amino acid transporter 1 | P43003 | 0.50 | 0.62 | 1.24 |
| 55 | Endoplasmin | P14625 | 1.17 | 1.45 | 1.24 |
| 56 | Alpha-1-antichymotrypsin | P01011 | 1.51 | 1.86 | 1.23 |
| 57 | Tubulin alpha-1C chain | Q9BQE3 | 0.44 | 0.53 | 1.22 |
| 58 | Guanine nucleotide-binding protein G(I)/G(S)/G(T) subunit beta-2 | P62879 | 0.37 | 0.45 | 1.22 |
| 59 | Keratin, type I cytoskeletal 9 | P35527 | 1.26 | 1.53 | 1.22 |
| 60 | Proteasome subunit alpha type-7 | O14818 | 0.88 | 1.07 | 1.21 |
| 61 | Glutamine synthetase | P15104 | 0.57 | 0.67 | 1.18 |
| 62 | Ubiquitin carboxyl-terminal hydrolase 5 | P45974 | 1.04 | 1.23 | 1.18 |
| 63 | Aspartate aminotransferase, mitochondrial | P00505 | 0.81 | 0.95 | 1.17 |
| 64 | Heat shock protein HSP 90-alpha | P07900 | 0.71 | 0.83 | 1.17 |
| 65 | Microtubule-associated protein 4 | P27816 | 1.45 | 1.69 | 1.16 |
| 66 | Dynamin-1 | Q05193 | 0.41 | 0.48 | 1.16 |
| 67 | Phosphatidylethanolamine-binding protein 1 | P30086 | 0.55 | 0.64 | 1.15 |
| 68 | Collagen alpha-3(VI) chain | P12111 | 2.42 | 2.78 | 1.15 |
| 69 | Plasma protease C1 inhibitor | P05155 | 1.47 | 1.68 | 1.15 |
| 70 | Plectin | Q15149 | 0.95 | 1.09 | 1.14 |
| 71 | Spectrin beta chain, non-erythrocytic 1 | Q01082 | 0.55 | 0.63 | 1.14 |
| 72 | NSFL1 cofactor p47 | Q9UNZ2 | 1.06 | 1.20 | 1.14 |
| 73 | Cytosolic non-specific dipeptidase | Q96KP4 | 1.17 | 1.32 | 1.12 |
| 74 | Pyruvate dehydrogenase E1 component subunit beta, mitochondrial | P11177 | 0.63 | 0.70 | 1.12 |
| 75 | Annexin A6 | P08133 | 0.99 | 1.11 | 1.12 |
| 76 | Brain acid soluble protein 1 | P80723 | 0.34 | 0.38 | 1.11 |
| 77 | Ig kappa chain C region | P01834 | 1.90 | 2.11 | 1.11 |
| 78 | Carbonic anhydrase 2 | P00918 | 1.00 | 1.10 | 1.10 |
| 79 | 4-aminobutyrate aminotransferase, mitochondrial | P80404 | 0.75 | 0.83 | 1.10 |
| 80 | Filamin-A | P21333 | 1.69 | 1.85 | 1.10 |
| 81 | WD repeat-containing protein 1 | O75083 | 1.54 | 1.69 | 1.10 |
| 82 | Ras-related protein Rab-11B | Q15907 | 1.04 | 1.14 | 1.09 |
| 83 | T-complex protein 1 subunit epsilon | P48643 | 1.04 | 1.12 | 1.08 |
| 84 | Matrin-3 | P43243 | 0.84 | 0.90 | 1.08 |
| 85 | Stathmin | P16949 | 0.75 | 0.81 | 1.08 |
| 86 | Clusterin | P10909 | 1.18 | 1.27 | 1.08 |
| 87 | Neuroblast differentiation-associated protein AHNAK | Q09666 | 2.16 | 2.31 | 1.07 |
| 88 | Prelamin-A/C | P02545 | 1.67 | 1.80 | 1.07 |
| 89 | N(G),N(G)-dimethylarginine dimethylaminohydrolase 1 | O94760 | 0.64 | 0.68 | 1.07 |
| 90 | Polyubiquitin-C | P0CG48 | 1.08 | 1.15 | 1.07 |
| 91 | Band 3 anion transport protein | P02730 | 1.80 | 1.93 | 1.07 |
| 92 | Calnexin | P27824 | 1.34 | 1.44 | 1.07 |
| 93 | Protein disulfide-isomerase | P07237 | 2.10 | 2.24 | 1.07 |
| 94 | Talin-1 | Q9Y490 | 1.56 | 1.66 | 1.07 |
| 95 | Serpin H1 | P50454 | 2.99 | 3.17 | 1.06 |
| 96 | Nestin | P48681 | 3.40 | 3.62 | 1.06 |
| 97 | Contactin-1 | Q12860 | 0.33 | 0.35 | 1.06 |
| 98 | Dihydropyrimidinase-related protein 2 | Q16555 | 0.83 | 0.88 | 1.06 |
| 99 | 60S acidic ribosomal protein P2 | P05387 | 1.51 | 1.59 | 1.05 |
| 100 | Fatty acid-binding protein, brain | O15540 | 1.67 | 1.75 | 1.05 |
| 101 | Glyceraldehyde-3-phosphate dehydrogenase | P04406 | 1.07 | 1.12 | 1.05 |
| 102 | Protein disulfide-isomerase A6 | Q15084 | 1.34 | 1.40 | 1.05 |
| 103 | Complement C3 | P01024 | 1.59 | 1.66 | 1.05 |
| 104 | Vimentin | P08670 | 2.11 | 2.19 | 1.04 |
| 105 | Nucleophosmin | P06748 | 1.62 | 1.68 | 1.03 |
| 106 | Fibronectin | P02751 | 1.54 | 1.59 | 1.03 |
| 107 | Peroxiredoxin-5, mitochondrial | P30044 | 0.60 | 0.62 | 1.03 |
| 108 | Protein disulfide-isomerase A4 | P13667 | 3.03 | 3.11 | 1.02 |
| 109 | 14-3-3 protein epsilon | P62258 | 0.77 | 0.79 | 1.02 |
| 110 | Prohibitin-2 | Q99623 | 0.75 | 0.77 | 1.02 |
| 111 | Aconitate hydratase, mitochondrial | Q99798 | 1.18 | 1.20 | 1.02 |
| 112 | Fibrinogen alpha chain | P02671 | 1.62 | 1.65 | 1.02 |
| 113 | Antithrombin-III | P01008 | 1.48 | 1.50 | 1.02 |
| 114 | UMP-CMP kinase | P30085 | 0.84 | 0.86 | 1.01 |
| 115 | Septin-11 | Q16181 | 0.63 | 0.63 | 1.00 |
| 116 | Apolipoprotein A-II | P02652 | 1.79 | 1.78 | 1.00 |
| 117 | Gelsolin | P06396 | 1.47 | 1.46 | 0.99 |
| 118 | Acetyl-CoA acetyltransferase, mitochondrial | P24752 | 1.17 | 1.16 | 0.99 |
| 119 | Fructose-bisphosphate aldolase C | P09972 | 0.66 | 0.65 | 0.98 |
| 120 | Microtubule-associated protein 1B | P46821 | 0.96 | 0.94 | 0.98 |
| 121 | Sodium/potassium-transporting ATPase subunit beta-1 | P05026 | 0.52 | 0.51 | 0.98 |
| 122 | Keratin, type II cytoskeletal 2 epidermal | P35908 | 1.37 | 1.33 | 0.98 |
| 123 | Glucose-6-phosphate isomerase | P06744 | 1.04 | 1.01 | 0.98 |
| 124 | Protein disulfide-isomerase A3 | P30101 | 1.30 | 1.27 | 0.98 |
| 125 | POTE ankyrin domain family member E | Q6S8J3 | 1.25 | 1.22 | 0.97 |
| 126 | Cathepsin D | P07339 | 1.15 | 1.12 | 0.97 |
| 127 | Cystatin-B | P04080 | 1.14 | 1.11 | 0.97 |
| 128 | Transitional endoplasmic reticulum ATPase | P55072 | 1.44 | 1.39 | 0.97 |
| 129 | Succinyl-CoA | P55809 | 0.74 | 0.72 | 0.97 |
| 130 | Creatine kinase B-type | P12277 | 0.47 | 0.45 | 0.96 |
| 131 | Alpha-synuclein | P37840 | 0.50 | 0.48 | 0.96 |
| 132 | ATP synthase subunit alpha, mitochondrial | P25705 | 0.63 | 0.60 | 0.95 |
| 133 | Cullin-associated NEDD8-dissociated protein 1 | Q86VP6 | 1.28 | 1.22 | 0.95 |
| 134 | Neuromodulin | P17677 | 0.72 | 0.69 | 0.95 |
| 135 | Cytochrome c oxidase subunit 5B, mitochondrial | P10606 | 0.57 | 0.54 | 0.94 |
| 136 | Synaptotagmin-1 | P21579 | 0.33 | 0.31 | 0.94 |
| 137 | Elongation factor 2 | P13639 | 2.42 | 2.27 | 0.94 |
| 138 | Alpha-1B-glycoprotein | P04217 | 1.00 | 0.94 | 0.94 |
| 139 | Phosphoglycerate mutase 1 | P18669 | 0.79 | 0.73 | 0.93 |
| 140 | Nicotinamide phosphoribosyltransferase | P43490 | 2.10 | 1.95 | 0.93 |
| 141 | Flavin reductase (NADPH) | P30043 | 1.18 | 1.09 | 0.92 |
| 142 | Heat shock cognate 71 kDa protein | P11142 | 1.02 | 0.94 | 0.92 |
| 143 | Heterogeneous nuclear ribonucleoprotein U | Q00839 | 1.34 | 1.23 | 0.92 |
| 144 | Superoxide dismutase [Mn], mitochondrial | P04179 | 2.33 | 2.14 | 0.92 |
| 145 | Heterogeneous nuclear ribonucleoprotein H2 | P55795 | 1.64 | 1.50 | 0.91 |
| 146 | Carbonyl reductase [NADPH] 1 | P16152 | 1.17 | 1.06 | 0.91 |
| 147 | Heterogeneous nuclear ribonucleoprotein D0 | Q14103 | 1.29 | 1.17 | 0.91 |
| 148 | Acyl-CoA-binding protein | P07108 | 1.57 | 1.43 | 0.91 |
| 149 | Peroxiredoxin-1 | Q06830 | 1.25 | 1.13 | 0.90 |
| 150 | Rab GDP dissociation inhibitor alpha | P31150 | 0.78 | 0.70 | 0.90 |
| 151 | F-actin-capping protein subunit alpha-2 | P47755 | 1.26 | 1.13 | 0.90 |
| 152 | Transgelin-2 | P37802 | 2.12 | 1.90 | 0.90 |
| 153 | Ubiquitin carboxyl-terminal hydrolase isozyme L1 | P09936 | 0.53 | 0.47 | 0.90 |
| 154 | Prohibitin | P35232 | 1.11 | 0.99 | 0.89 |
| 155 | ATP synthase subunit beta, mitochondrial | P06576 | 0.67 | 0.60 | 0.89 |
| 156 | Tubulin polymerization-promoting protein | O94811 | 0.44 | 0.39 | 0.89 |
| 157 | Transketolase | P29401 | 1.64 | 1.46 | 0.89 |
| 158 | Adenylyl cyclase-associated protein 1 | Q01518 | 1.61 | 1.43 | 0.89 |
| 159 | Ras-related protein Rab-7a | P51149 | 2.16 | 1.91 | 0.89 |
| 160 | Nucleolin | P19338 | 2.07 | 1.83 | 0.89 |
| 161 | Protein DJ-1 | Q99497 | 0.81 | 0.71 | 0.88 |
| 162 | 60S acidic ribosomal protein P0 | P05388 | 1.62 | 1.41 | 0.87 |
| 163 | Cofilin-1 | P23528 | 0.97 | 0.84 | 0.87 |
| 164 | Galectin-1 | P09382 | 1.61 | 1.40 | 0.87 |
| 165 | Aldehyde dehydrogenase, mitochondrial | P05091 | 0.81 | 0.70 | 0.87 |
| 166 | Isocitrate dehydrogenase [NADP], mitochondrial | P48735 | 0.85 | 0.73 | 0.86 |
| 167 | Transaldolase | P37837 | 1.17 | 1.00 | 0.86 |
| 168 | Ubiquitin-like modifier-activating enzyme 1 | P22314 | 1.20 | 1.02 | 0.85 |
| 169 | Puromycin-sensitive aminopeptidase | P55786 | 1.44 | 1.22 | 0.85 |
| 170 | A-kinase anchor protein 12 | Q02952 | 1.36 | 1.14 | 0.84 |
| 171 | Glycogen phosphorylase, brain form | P11216 | 1.49 | 1.26 | 0.84 |
| 172 | Reticulon-4 | Q9NQC3 | 1.56 | 1.31 | 0.84 |
| 173 | Alpha-enolase | P06733 | 1.18 | 0.98 | 0.84 |
| 174 | Stress-70 protein, mitochondrial | P38646 | 0.89 | 0.73 | 0.83 |
| 175 | Adenylate kinase isoenzyme 1 | P00568 | 0.85 | 0.71 | 0.83 |
| 176 | Fumarate hydratase, mitochondrial | P07954 | 1.20 | 0.99 | 0.83 |
| 177 | Fibrinogen gamma chain | P02679 | 2.83 | 2.34 | 0.83 |
| 178 | Clathrin heavy chain 1 | Q00610 | 1.87 | 1.54 | 0.83 |
| 179 | L-lactate dehydrogenase A chain | P00338 | 1.82 | 1.49 | 0.82 |
| 180 | L-lactate dehydrogenase B chain | P07195 | 1.16 | 0.95 | 0.82 |
| 181 | Annexin A5 | P08758 | 2.68 | 2.19 | 0.82 |
| 182 | Apolipoprotein A-I | P02647 | 2.56 | 2.07 | 0.81 |
| 183 | Vinculin | P18206 | 1.93 | 1.55 | 0.80 |
| 184 | Triosephosphate isomerase | P60174 | 1.12 | 0.90 | 0.80 |
| 185 | 60 kDa heat shock protein, mitochondrial | P10809 | 1.07 | 0.86 | 0.80 |
| 186 | Syntaxin-1B | P61266 | 0.29 | 0.23 | 0.80 |
| 187 | Secernin-1 | Q12765 | 0.64 | 0.51 | 0.79 |
| 188 | Elongation factor 1-alpha 1 | Q5VTE0 | 1.76 | 1.38 | 0.79 |
| 189 | Glutathione S-transferase P | P09211 | 1.60 | 1.25 | 0.78 |
| 190 | Voltage-dependent anion-selective channel protein 1 | P21796 | 0.51 | 0.39 | 0.78 |
| 191 | Pyruvate kinase isozymes M1/M2 | P14618 | 1.65 | 1.28 | 0.77 |
| 192 | Alpha-crystallin B chain | P02511 | 1.64 | 1.27 | 0.77 |
| 193 | Glial fibrillary acidic protein | P14136 | 1.91 | 1.47 | 0.77 |
| 194 | Proteasome subunit beta type-1 | P20618 | 1.36 | 1.05 | 0.77 |
| 195 | Angiotensinogen | P01019 | 1.49 | 1.14 | 0.77 |
| 196 | Annexin A2 | P07355 | 2.40 | 1.85 | 0.77 |
| 197 | Fructose-bisphosphate aldolase A | P04075 | 1.03 | 0.80 | 0.77 |
| 198 | Chitinase-3-like protein 1 | P36222 | 2.38 | 1.83 | 0.77 |
| 199 | Stress-induced-phosphoprotein 1 | P31948 | 1.12 | 0.86 | 0.77 |
| 200 | Sorcin | P30626 | 1.68 | 1.29 | 0.77 |
| 201 | Elongation factor 1-delta | P29692 | 1.75 | 1.34 | 0.76 |
| 202 | Heterogeneous nuclear ribonucleoproteins A2/B1 | P22626 | 1.47 | 1.13 | 0.76 |
| 203 | Phosphoglucomutase-1 | P36871 | 1.62 | 1.23 | 0.76 |
| 204 | RNA-binding motif protein, X chromosome | P38159 | 2.08 | 1.57 | 0.75 |
| 205 | Far upstream element-binding protein 2 | Q92945 | 2.98 | 2.23 | 0.75 |
| 206 | Neural cell adhesion molecule 1 | P13591 | 0.52 | 0.38 | 0.74 |
| 207 | Profilin-1 | P07737 | 2.19 | 1.62 | 0.74 |
| 208 | Ubiquitin-conjugating enzyme E2 N | P61088 | 0.84 | 0.62 | 0.74 |
| 209 | Transthyretin | P02766 | 1.99 | 1.44 | 0.72 |
| 210 | ES1 protein homolog, mitochondrial | P30042 | 1.15 | 0.83 | 0.72 |
| 211 | Elongation factor 1-gamma | P26641 | 2.25 | 1.62 | 0.72 |
| 212 | Malate dehydrogenase, mitochondrial | P40926 | 0.87 | 0.62 | 0.72 |
| 213 | Gamma-synuclein | O76070 | 0.39 | 0.28 | 0.71 |
| 214 | Actin-related protein 2 | P61160 | 2.01 | 1.42 | 0.71 |
| 215 | 6-phosphogluconolactonase | O95336 | 2.21 | 1.56 | 0.70 |
| 216 | Keratin, type I cytoskeletal 10 | P13645 | 1.20 | 0.83 | 0.70 |
| 217 | Heat shock protein beta-1 | P04792 | 2.02 | 1.40 | 0.69 |
| 218 | Peroxiredoxin-6 | P30041 | 1.49 | 1.02 | 0.69 |
| 219 | Trifunctional enzyme subunit beta, mitochondrial | P55084 | 1.43 | 0.97 | 0.68 |
| 220 | Voltage-dependent anion-selective channel protein 2 | P45880 | 0.39 | 0.27 | 0.68 |
| 221 | Adenosylhomocysteinase | P23526 | 2.04 | 1.39 | 0.68 |
| 222 | Proteasome activator complex subunit 2 | Q9UL46 | 4.95 | 3.36 | 0.68 |
| 223 | T-complex protein 1 subunit gamma | P49368 | 1.32 | 0.89 | 0.68 |
| 224 | ADP/ATP translocase 3 | P12236 | 0.78 | 0.52 | 0.67 |
| 225 | Alpha-actinin-1 | P12814 | 2.65 | 1.77 | 0.67 |
| 226 | Retinal dehydrogenase 1 | P00352 | 1.30 | 0.84 | 0.65 |
| 227 | Cytosol aminopeptidase | P28838 | 2.26 | 1.45 | 0.64 |
| 228 | Peptidyl-prolyl cis-trans isomerase B | P23284 | 2.48 | 1.57 | 0.63 |
| 229 | Nucleoside diphosphate kinase B | P22392 | 1.68 | 1.06 | 0.63 |
| 230 | Peptidyl-prolyl cis-trans isomerase A | P62937 | 1.04 | 0.64 | 0.62 |
| 231 | Transgelin | Q01995 | 2.31 | 1.38 | 0.60 |
| 232 | Phosphoglycerate kinase 1 | P00558 | 1.63 | 0.98 | 0.60 |
| 233 | Myristoylated alanine-rich C-kinase substrate | P29966 | 1.27 | 0.76 | 0.59 |
| 234 | Annexin A1 | P04083 | 2.92 | 1.73 | 0.59 |
| 235 | Tenascin-R | Q92752 | 0.98 | 0.58 | 0.59 |
| 236 | X-ray repair cross-complementing protein 6 | P12956 | 2.28 | 1.29 | 0.56 |
| 237 | Fibrinogen beta chain | P02675 | 2.79 | 1.33 | 0.48 |
| 238 | Polypyrimidine tract-binding protein 1 | P26599 | 1.91 | 0.90 | 0.47 |
| 239 | Histone H3.1t | Q16695 | 3.65 | 1.62 | 0.44 |
| 240 | Histone H2B type 1-M | Q99879 | 2.36 | 1.04 | 0.44 |
| 241 | Protein dpy-30 homolog | Q9C005 | 2.31 | 1.01 | 0.44 |
| 242 | Protein S100-A9 | P06702 | 3.92 | 1.65 | 0.42 |
| 243 | X-ray repair cross-complementing protein 5 | P13010 | 2.01 | 0.83 | 0.41 |

**Supplementary Table s4. Common proteins identified in serum and tissue proteomic analysis of SVZ+ and SVZ- GBMs using iTRAQ and 2D-DIGE methods**

| **Serum vs. Tissue proteomic comparison of SVZ+/SVZ-** | | | |
| --- | --- | --- | --- |
| Accession | Protein Name | Serum SVZ+/SVZ-_iTRAQ | Tissue SVZ+/SVZ-_iTRAQ |
| P02763 | Alpha-1-acid glycoprotein 1 | 1.20 | 2.78 |
| P01011 | Alpha-1-antichymotrypsin | 1.14 | 1.23 |
| P01009 | Alpha-1-antitrypsin | 0.99 | 1.93 |
| P04217 | Alpha-1B-glycoprotein | 0.90 | 0.94 |
| P01023 | Alpha-2-macroglobulin | 0.93 | 1.48 |
| P01008 | Antithrombin-III | 0.94 | 1.02 |
| P02647 | Apolipoprotein A-I | 0.91 | 0.81 |
| P02652 | Apolipoprotein A-II | 0.80 | 1.00 |
| P10909 | Clusterin | 1.10 | 1.08 |
| P01024 | Complement C3 | 0.99 | 1.05 |
| P00738 | Haptoglobin | 0.86 | 1.82 |
| P69905 | Hemoglobin subunit alpha | 1.03 | 1.70 |
| P68871 | Hemoglobin subunit beta | 0.39 | 1.90 |
| P01871 | Ig mu chain C region | 1.07 | 1.31 |
| P02766 | Transthyretin | 0.90 | 0.72 |
| P02774 | Vitamin D-binding protein | 1.59 | 1.70 |
| **Serum vs Tissue (DIGE vs iTRAQ)** | | | |
| Accession | Protein Name | Serum SVZ+/SVZ- _2D-DIGE | Tissue SVZ+/SVZ-_iTRAQ |
| P02768 | Serum albumin | 1.68 | 1.47 |
| P01011 | Alpha-1-antichymotrypsin | -1.58 | 1.23 |
| P02647 | Apolipoprotein A-I | -1.82 | -1.23 |
| **Serum (iTRAQ vs 2D-DIGE)** | | | |
| Accession | Protein Name | Serum SVZ+/SVZ- (iTRAQ) | Serum SVZ+/SVZ- (2D-DIGE) |
| P02768 | Serum Albumin | -1.1 | 1.68 |
| P01011 | α-1-antichymotrypsin | 1.14 | -1.58 |
| P02790 | Hemopexin | 1.11 | 1.21 |
| P02647 | Apolipoprotein A-I | -1.1 | -1.82 |
| **Tissue (iTRAQ vs 2D-DIGE)** | | | |
| Accession | Protein Name | Tissue SVZ+/SVZ- (iTRAQ) | Tissue SVZ+/SVZ- (2D-DIGE) |
| P31150 | Rab GDP dissociation inhibitor alpha | -1.11 | 2.1 |
| P08670 | Vimentin | 1.04 | 2.4 |

**Supplementary Table s5. Correlation of iTRAQ-based proteomics data (SVZ+ vs SVZ- GBMs) with TCGA gene expression data (Short-term survivors vs Long-term survivors)**

| S.L. No. | Entry_name | Accession number | iTRAQ data Fold change  (SVZ+/SVZ-) | TCGA Data  mRNA expression Fold-change (STS/LTS) |
| --- | --- | --- | --- | --- |
| 1 | Brain acid soluble protein 1 | P80723 | 1.11 | 0.59 |
| 2 | Aspartate aminotransferase, cytoplasmic | P17174 | 1.41 | 0.79 |
| **3** | **RNA-binding motif protein, X chromosome** | **P38159** | **0.75** | **0.85** |
| **4** | **Adenosylhomocysteinase** | **P23526** | **0.68** | **0.86** |
| 5 | Keratin, type I cytoskeletal 10 | P13645 | 0.70 | 1.16 |
| **6** | **Protein disulfide-isomerase** | **P07237** | **1.07** | **1.16** |
| **7** | **Vimentin** | **P08670** | **1.04** | **1.17** |
| 8 | Annexin A5 | P08758 | 0.82 | 1.21 |
| 9 | Glial fibrillary acidic protein | P14136 | 0.77 | 1.22 |
| 10 | Peptidyl-prolyl cis-trans isomerase B | P23284 | 0.63 | 1.23 |
| **11** | **Endoplasmin** | **P14625** | **1.24** | **1.24** |
| **12** | **Dihydropyrimidinase-related protein 2** | **Q16555** | **1.06** | **1.26** |
| **13** | **Glutamine synthetase** | **P15104** | **1.18** | **1.27** |
| 14 | Secernin-1 | Q12765 | 0.79 | 1.30 |
| 15 | Cytosol aminopeptidase | P28838 | 0.64 | 1.30 |
| 16 | L-lactate dehydrogenase A chain | P00338 | 0.82 | 1.31 |
| **17** | **T-complex protein 1 subunit zeta** | **P40227** | **1.31** | **1.32** |
| 18 | Transgelin-2 | P37802 | 0.90 | 1.32 |
| **19** | **Fibronectin** | **P02751** | **1.03** | **1.34** |
| 20 | Galectin-1 | P09382 | 0.87 | 1.38 |
| 21 | Annexin A2 | P07355 | 0.77 | 1.38 |
| 22 | Microtubule-associated protein 1B | P46821 | 0.98 | 1.39 |
| 23 | Annexin A1 | P04083 | 0.59 | 1.42 |
| **24** | **Complement C3** | **P01024** | **1.05** | **1.44** |
| 25 | Protein S100-A9 | P06702 | 0.42 | 1.45 |
| **26** | **Ceruloplasmin** | **P00450** | **1.48** | **1.47** |
| 27 | Alpha-actinin-1 | P12814 | 0.67 | 1.49 |
| 28 | Carbonyl reductase [NADPH] 1 | P16152 | 0.91 | 1.51 |
| **29** | **Alpha-2-macroglobulin** | **P01023** | **1.48** | **1.52** |
| 30 | Superoxide dismutase [Mn], mitochondrial | P04179 | 0.92 | 1.56 |
| **31** | **Clusterin** | **P10909** | **1.08** | **1.57** |
| 32 | Transgelin | Q01995 | 0.60 | 1.63 |
| **33** | **Tenascin** | **P24821** | **1.47** | **1.64** |
| 34 | Angiotensinogen | P01019 | 0.77 | 1.68 |
| **35** | **Moesin** | **P26038** | **1.25** | **1.71** |
| 36 | Neuromodulin | P17677 | 0.95 | 1.73 |
| 37 | A-kinase anchor protein 12 | Q02952 | 0.84 | 1.86 |
| **38** | **Excitatory amino acid transporter 1** | **P43003** | **1.24** | **1.91** |
| **39** | **Alpha-1-antichymotrypsin** | **P01011** | **1.23** | **2.19** |
| **40** | **Plasma protease C1 inhibitor** | **P05155** | **1.15** | **2.26** |
| **41** | **Fatty acid-binding protein, brain** | **O15540** | **1.05** | **2.64** |
| 42 | Chitinase-3-like protein 1 | P36222 | 0.77 | 4.29 |

**Note: Proteins/genes exhibiting similar expression trends were highlighted in bold.**

**Supplementary Table s6. Pathways found to be affected in SVZ- GBM tumors (with respect to peritumoral tissue controls)**

| Category | Term | Count | % | P-Value |
| --- | --- | --- | --- | --- |
| KEGG_PATHWAY | Parkinson's disease | 8 | 6.1 | 2.20E-03 |
| KEGG_PATHWAY | Huntington's disease | 8 | 6.1 | 1.40E-02 |
| KEGG_PATHWAY | Focal adhesion | 7 | 5.3 | 6.70E-02 |
| KEGG_PATHWAY | Glycolysis / Gluconeogenesis | 6 | 4.6 | 1.60E-03 |
| KEGG_PATHWAY | Oxidative phosphorylation | 6 | 4.6 | 3.80E-02 |
| KEGG_PATHWAY | Alzheimer's disease | 6 | 4.6 | 8.40E-02 |
| KEGG_PATHWAY | Complement and coagulation cascades | 5 | 3.8 | 1.70E-02 |
| KEGG_PATHWAY | Pentose phosphate pathway | 4 | 3.1 | 5.20E-03 |
| KEGG_PATHWAY | PPAR signaling pathway | 4 | 3.1 | 7.60E-02 |
| KEGG_PATHWAY | Cysteine and methionine metabolism | 3 | 2.3 | 8.50E-02 |

**Supplementary Table s7. Pathways found to be affected in SVZ+ GBM tumors (with respect to peritumoral tissue controls)**

| Category | Term | Count | % | P-Value |
| --- | --- | --- | --- | --- |
| KEGG_PATHWAY | Parkinson's disease | 9 | 7.8 | 1.20E-04 |
| KEGG_PATHWAY | Complement and coagulation cascades | 7 | 6.1 | 1.50E-04 |
| KEGG_PATHWAY | Huntington's disease | 8 | 7 | 5.40E-03 |
| KEGG_PATHWAY | Focal adhesion | 8 | 7 | 9.70E-03 |
| KEGG_PATHWAY | Pentose phosphate pathway | 3 | 2.6 | 3.60E-02 |
| KEGG_PATHWAY | Regulation of actin cytoskeleton | 7 | 6.1 | 4.30E-02 |
| KEGG_PATHWAY | Alzheimer's disease | 6 | 5.2 | 4.50E-02 |
| KEGG_PATHWAY | ECM-receptor interaction | 4 | 3.5 | 7.90E-02 |

**Supplementary Table s8. Pathways found to be affected in SVZ+ GBM tumors (with respect to SVZ- GBMs)**

| Category | Term | Count | % | P-Value |
| --- | --- | --- | --- | --- |
| KEGG_PATHWAY | Vibrio cholerae infection | 3 | 5.3 | 5.30E-02 |
| KEGG_PATHWAY | Tight junction | 4 | 7 | 5.90E-02 |
| KEGG_PATHWAY | Non-homologous end-joining | 2 | 3.5 | 8.40E-02 |

**Supplementary Table s9.** ELISA quantification for serum hemopexin in SVZ+ & SVZ- GBM patients

| **SVZ+ Sample** | **Conc. (mg/ml)** | **SVZ- Sample** | **Conc.(mg/ml)** |
| --- | --- | --- | --- |
| Patient 1 | 1.92 | Patient 1 | 1.03 |
| Patient 2 | 1.95 | Patient 2 | 1.67 |
| Patient 3 | 1.49 | Patient 3 | 1.32 |
| Patient 4 | 1.64 | Patient 4 | 1.35 |
| Patient 5 | 2.06 | Patient 5 | 1.12 |
| Patient 6 | 0.99 | Patient 6 | 1.34 |
| Patient 7 | 1.41 | Patient 7 | 0.96 |
| Patient 8 | 2.43 | Patient 8 | 0.89 |
| Patient 9 | 1.87 | Patient 9 | 1.38 |
| Patient 10 | 1.39 | Patient 10 | 1.47 |
| Mean = 1.7155 | | Mean = 1.253 | |
| Median = 1.755 | | Median = 1.33 | |
| Fold change (SVZ+/ SVZ-) = 1.368715084 | | | |
| *p* value (*t-*test): 0.0070(<0.05) | | | |

**Supplementary materials and methods**

**Patient selection and samples collection**

GBM serum and tissue samples were collected from Tata Memorial Centre, Advanced Centre for Treatment, Research and Education (TMC-ACTREC), Mumbai, India. The tissue and serum samples were categorized into two groups based on sub-ventricular zone involvement and median age of the patients. Total 21 tissue samples (9 SVZ-, 8 SVZ+ and 4 peritumoral tissues) and 33 serum samples (16 SVZ- and 17 SVZ+) were used in the current proteomics study. A comparative proteomics analysis was carried out after approval of the institutional ethics committee of TMH and IIT Bombay. Peritumoral tissue samples were used as normal control samples.

**Serum samples processing**

Protein extraction from serum was performed using TCA –Acetone precipitation method^65^. Crude serum samples were diluted 5 times in phosphate buffer (pH 7.4) and then subjected to sonication (8 cycles of 20% amplitude, 5 sec pulse and 30 sec gap).

Two high abundant serum proteins, albumin (50-60%) and Immunoglobulin IgG (15-25%) were depleted using Albumin & IgG Depletion Spin Trap columns (GE Healthcare) as per the manufacturer’s instructions. The serum proteins were precipitated by adding 4 volumes of ice-cold TCA-Acetone (10 % w/v) to the depleted serum sample. The serum samples were then briefly vortexed and incubated at -20̊ °C for 2 hours to precipitate the serum proteins, followed by centrifugation at 15000g for 20 minutes at 4̊ °C. Protein pellets were air- dried and dissolved in the rehydration buffer, containing 8M urea, 2 M thiourea, CHAPS (4% w/v), 40 mM DTT, IPG buffer (2% v/v; pH 4-7; Linear) and Bromophenol blue (0.002 %). The extracted serum protein samples were cleaned up using 2D Clean up kit (GE Healthcare) following the manufacturer’s instructions.

**Protein extraction from SVZ+ and SVZ- GBM tissues**

50 mg of tumor tissue was weighed and used for protein extraction. Protein extraction was performed using trizol method^66^. In brief, 0.5 ml of Phosphate buffer saline (PBS) was added to the weighed tissue and sonicated at 40% amplitude, 5 sec pulse on and off for 2 min and 30 sec. The cell lysate so obtained was subjected to centrifugation at 12,000 rpm for 10minutes to pellet down the cell debris, leaving behind the supernatant containing proteins. The supernatant was transferred to a fresh tube followed by addition of 1ml and 0.2 ml of trizol and chloroform, respectively. The resultant mixture was vortexed and placed on ice for phase separation following which the sample was centrifuged at 14,000 rpm and 4 °C for 15 min to separate the sample into three phases. The top aqueous phase containing RNA was discarded, while the interphase and the lower layer organic phase were added with 0.3 ml of absolute ethanol and placed on ice for 5 min. The tube was then centrifuged at 2,000 rpm for 5 min to pellet down genomic DNA. To the supernatant, 4 volumes of chilled acetone was added and the tube was kept at -20 °C for 2 hours followed by centrifugation at 14,000 rpm and 4 °C for 30 min. The pellet obtained was dispersed by briefly sonicating in 0.3M Guanidinium hydrochloride - 95% ethanol to remove any trizol that was bound to the pellet. This washing step was performed thrice, followed by washing with chilled acetone. After acetone washes, the pellet was air-dried to remove acetone and dissolved in rehydration buffer. The protein samples were quantified using 2D-Quant kit (GE Healthcare) and the protein samples were stored at -20 °C till use for labeling or buffer exchange.

**Two dimensional difference gel electrophoresis (2D-DIGE)**

The protein samples in rehydration buffer were minimal labeled with CyDyes as per the manufacturer's instructions (GE Healthcare). To ensure optimum labeling, the pH of the samples was set to 8.5 using 100 mM NaOH. The SVZ+ and SVZ- GBM protein samples were alternately labeled using Cy3 and Cy5 to negate variations, if any, due to the dyes while a pool of all the 12 individual samples (16 individuals samples in case of serum analysis) was used as an internal standard and labeled using Cy2. 50 µg of the tissue protein sample (60 µg of serum) was labeled with 400 pmol of CyDye by incubating the tube on ice for 60minutes in dark. The excess dye that remained after incubation was quenched by adding 10mM lysine followed by incubation on ice for 10 min in dark. The labeled protein samples were then pooled and the volume was made up to 350 µL with rehydration buffer and loaded onto an 18 cm IPG strip of pH 4-7 (linear). The entire process of rehydration lasted for 14 hours following which the rehydrated strips were isoelectrically focused using Ettan IPGphor 3 instrument (GE Healthcare) for a total of ~ 80,000 Vh. The IEF settings for tissue protein samples was as follows: 200 V for 4 h, 500 V for 1 h, 1000 V gradient for 750 Vh, 8000 V gradient for 13,500 Vh, 8000 V step for 64,000 Vh. and a holding step of 500 V for 4 h.

The following IEF parameters were used for separation of serum proteins on IPG strip: 200 Volts for 4 h [step and hold], 500 Volts for 1 h [step and hold], 1000 Volts for 1 h [step and hold], 8000 Volts for 6 h [gradient], 8000 Volts for 5 h [step and hold] and 200 Volts for 5 h [step and hold]. The maximum limit for the current was set to 50 μA per strip.

The isoelectrically focused IPG strips were now equilibrated for 15minutes each in equilibration solution-1 and equilibration solution-2 prior to separation along the second dimension. Equilibration solution-1 containing 6 M Urea, 75 mM Tris-HCl pH 8.8, 29.3% (v/v) glycerol, 2% (w/v) SDS, and 0.002% (w/v) bromophenol blue and 1% (w/v) Dithiothreitol DTT reduces the disulfide bonds while equilibration solution-2 containing 6 M urea, 75 mM Tris-HCl pH 8.8, 29.3% (v/v) glycerol, 2% (w/v) SDS, and 0.002% (w/v) bromophenol blue and 1% (w/v) iodoacetamide (IAA) helps in alkylating the thiol groups. The proteins separated isoelectrically on the strip were now subjected to SDS-PAGE on 12.5% gel in Ettan DALT electrophoresis unit (GE Healthcare) for further separation on the basis of molecular weight.

The entire process from labeling to electrophoretic run was performed in dark since the CyDyes are light sensitive.

After completion of SDS-PAGE, the gels were scanned using Typhoon FLA 9500 scanner (GE Healthcare) at different excitation and emission wavelengths. The excitation wavelengths for Cy2, Cy3 and Cy5 dyes are 488, 532 and 633 nm, respectively and their emission wavelengths are 520, 580 and 670 nm, respectively. All the images were scanned at 100 µm resolution and the images obtained were further processed using DeCyder 2D software version 7.0 (GE Healthcare).

**Image analysis**

2D-DIGE gels were analysed using DeCyder software to determine the relative quantitation of different brain tissue protein spots. While the proteome comparison between two individual patients was done using Differential in-gel analysis (DIA) module, the comparison of proteome of two different groups of patients was done using Biological variation analysis (BVA) module. (Each group had a set of gels representing the proteome of patients suffering from the same disease/grade of a disease). The protein spots with p value ≤ 0.05 were considered statistically significant.

**In-gel digestion**

To reveal the identity of the differentially expressed and statistically significant (*p* ≤ 0.05) protein spots identified from the 2D-DIGE experiments, mass spectrometric analysis was performed. Significant protein spots were manually excised from the 2D gels and subjected to in-gel digestion^67^. The protein spots were washed with 25mM ammonium bicarbonate (ABC) solution and solution-A (1:2 ratio of 25mM ABC: Acetonitrile) alternately. After addition of each solution, the gel pieces were vortexed for 5 min followed by discarding the solution. The washing step described above was repeated twice to completely remove the Coomassie brilliant blue dye. The proteins in the gel pieces were reduced by adding 10 mM dithiothreitol (DTT) and incubation at 56 °C in a dry bath for 1 hour. The gel pieces were washed with 25 mM ABC followed by alkylation using 50 mM iodoacetamide (IAA) and incubated in the dark (30 minutes) at room temperature. Now the gel pieces were washed using 25 mM ABC & solution-A as mentioned above and air dried. To each protein spot, 400 ng of trypsin was added and incubated on ice for 30 min. followed by addition of 50 µl 50 mM ABC and incubated at 37 °C for 16 hours. The peptides from the gel pieces were extracted using a gradient of 50%, 60% and 80% acetonitrile solution in 0.1% formic acid. The extracted peptides were concentrated using a SpeedVac before subjecting to mass spectrometry. The identity of the significantly altered serum protein spots was revealed by MALDI-TOF/TOF analysis, where as the significant protein spots from the tissue proteomic analysis (from 2D-DIGE method) were identified using Q Exactive Benchtop Orbitrap mass spectrometer (Thermo Fisher Scientific).

**MALDI-TOF/TOF analysis**

The peptide solution was mixed with the MALDI matrix i.e., α-cyano-4-hydroxy-cinnamic acid (CHCA) and spotted onto MALDI plate and air dried. The air dried spots were subjected to mass spectrometry using 4800 MALDI TOF/TOF MS (AB Sciex) in reflectron mode. The mass spectrometry data was analysed using MASCOT version 2.1 search engine for identification of the protein against Swiss-Prot database. Following parameters were used for the identification of proteins using mascot search engine: mass tolerance for MS was 75 ppm and for MS/MS 0.4 Da, fixed modification included carbamidomethylation at the Cysteine residues and variable modification included oxidation of methionine residues.

**Buffer exchange and in-solution digestion**

The brain tissue proteins in rehydration buffer and the depleted serum proteins were made both iTRAQ reagent and LC-MS compatible by exchanging in 0.5 M TEAB buffer (Tri Ethyl Ammonium Bicarbonate buffer). The 3 kDa molecular weight cut-off filters (MWCO) used for this were first wet with 0.5 M TEAB followed by adding 600 µg of pooled protein sample in rehydration buffer and making the volume up to 500 µL using 0.5 M TEAB buffer. The tubes were briefly vortexed before centrifugation at 10,000 rpm for 30 minutes at 4 °C. Molecules less than 3 kDa size moved out of the filter leaving behind the larger molecules. The volume was again made up to 500 µL by adding 0.5 M TEAB buffer and the whole process of brief vortexing and centrifugation was repeated 4-5 times. After this, the buffer exchanged protein solution was collected in a fresh tube and re-quantified using 2D- Quant kit (GE Healthcare). 100 µg of the protein solution was reduced using TCEP (tris-(2-carboxy ethyl) phosphine) by incubating at 65 °C for 1 hour followed by alkylation using IAA (Iodoacetamide). The reduced and alkylated protein solution was then in-solution digested using trypsin (1:20) at 37 °C for 16 hours.

**iTRAQ labeling**

iTRAQ labeling of the digested tissue protein samples was done following the manufacturer's instructions. The iTRAQ labels 114, 115 and 116 were reconstituted in 70 µL of absolute ethanol provided with the kit. The in-solution digested normal brain tissue protein, SVZ- and SVZ+ GBM tumor tissue protein samples were labeled with iTRAQ reagent 114, 115 and 116, respectively. In set one and two, the iTRAQ 117 reagent was used for labeling of in-solution digested SVZ- GBM tissue proteins, while in set three and four, the iTRAQ 117 reagent was used for labeling of in-solution digested SVZ+ GBM tumor tissue proteome. The tubes with the labels were then left for incubation at room temperature for 1 hour following which the reaction was stopped using 100 µL of Milli-Q water. Finally, the samples were pooled and subjected to off-gel fractionation.

In case of serum proteomic analysis SVZ- serum proteome was labeled with iTRAQ reagent 114 & 116, where as the SVZ+ GBM patient’s serum proteome was labeled with iTRAQ reagent 115 & 117. After labeling process, the reaction was quenched using Milli-Q water, and the labeled samples were pooled and subjected to off-gel fractionation.

**Offgel fractionation of the iTRAQ labeled samples**

200µg of the labeled sample was subjected to Off-gel fractionation on 3-10 non-linear, 24cm high resolution IPG strips using Agilent 3100 OffGel fractionator. Minor modifications were made to the manufacturer's protocol (Agilent Technologies) and followed for the experiment. The process of Off -Gel fractionation was initiated by swelling the IPG strips using 40 µL of Milli-Q water in each loading cup on the IPG strip and leaving it for 30 minutes. The in-solution digested protein sample volume was made upto 3.6 ml using Milli-Q and was distributed equally in each of the cups on the IPG strip. The default protocol in the instrument (Total volt hours = 50 kVh) was used for isoelectric focussing of peptides. On completion of IEF, the fractions were collected and dried using SpeedVac. followed by reconstituting the fractions in 0.1% formic acid which were then subjected to LC-MS/MS analysis.

**LC-MS/MS**

Brain tumor tissue proteome was analyzed using Agilent 6550 iFunnel Q-TOF (Agilent Technologies) equipped with Chip cube and interfaced with nanoflow LC system (Agilent Technologies). The fractions obtained after off-gel fractionation were subjected to LC-MS/MS, where the peptides were enriched on the C18 enrichment column and separated on a 75 µm x 43mm analytical/ separation column in the protein chip (Agilent HPLC-Chip: G4240-62001ZORBAX 300SB-C18) using a gradient mobile phase consisting of two different solvents 0.1% formic acid solution (Solvent A) and 90% acetonitrile (Solvent B) at a flow rate of 0.5 µl/min. The following gradient method was used for the separation of peptide on the chip over a period of 50 min: 0-3 min, 5-20% solvent B; 3-45 min, 20-45% solvent B; 45-50 min, 45-95% solvent B; nitrogen gas was maintained at 250 °C with a flow rate of 9 L/min. The mass range in the MS was 100-3200 (m/z), with a MS Scan rate of 5 spectra/ sec. Total 15 high intense peptide ions (peaks) having charge ≥ 2 from MS were selected for MS/MS. In MS/MS mode, the mass range was 50-3200 (m/z), with a MS/MS scan rate of 5 spectra/sec. The data was acquired in centroid mode. The MS/MS data was further analyzed using Spectrum mill software (Agilent Technologies). The mass spectrometry data was searched against SwissProt database using *Homosapiens* as taxonomy; carbamidomethylation (C) & iTRAQ (N-term, K) as fixed modifications and Oxidation of methionine as variable modification; precursor and product mass tolerance were 20 and 50 ppm respectively.

The serum iTRAQ labeled off-gel fractionated proteome samples were analyzed on Q Exactive Benchtop Orbitrap mass spectrometer (Thermo Scientific). The iTRAQ labeled serum peptides were enriched on the trap column (Acclaim PepMap 100 75 µm x 2 cm, nano viper, C18, 3 µm, 100 A°) and separated on an analytical column (Acclaim PepMap RSLC 50 µm x 15 cm, nano viper, C18, 2 µm, 100 A°) using two different solvents, containing 5% acetonitrile in 0.1% formic acid (Solvent A) and 95% acetonitrile in 0.1% formic acid (Solvent B) at a flow rate of 300 nL/min. The peptide samples were separated on the column for 90 min using the following gradient method. The change in the organic solvent composition (solvent B) with respect to time as follows, from 0 – 68 min 5-25% solvent B, from 68-73 min 25-35% solvent B, from 73-75 min 35-90% solvent B, from 75-78 min 90-90% solvent B, from 78-83 min 90-05% solvent B, from 83-90 min 05-05% solvent B. In MS the precursor ions were scanned in the mass range of 350-1,800 m/z, with a mass resolution of 70,000. Top 10 intense ions were further selected for MS/MS. MS/MS was performed at a resolution of 17,500 using normalized collision energy of 27. The MS/MS data was acquired in centroid mode and the data analysis was performed using Proteome Discoverer 1.4 (ThermoScientific). Sequest algorithm was used to search the MS data against the human SwissProt database; carbamidomethylation (C) & iTRAQ (N-term, K) as fixed modifications and Oxidation (M), deamidated (N, Q) and iTRAQ 4plex (K) as dynamic modifications; the precursor and fragment mass tolerance were 5 ppm and 0.2Da respectively.

**Bioinformatics analysis**

mRNA expression data for 558 GBM patients was downloaded from TCGA data portal (<https://gdc-portal.nci.nih.gov/>) and further classified into short-term survivors (STS), median-term survivors (MTS) and long-term survivors (LTS) based on their overall survival. GBM patients with survival of <1 year, 1-3 years and >3 years were classified as STS, MTS and LTS, respectively. Of the 558 GBM patients, only 38 patients had an overall survival (OS) of >3 years and 217 patients with an overall survival of <1 year. The mRNA expression of STS and LTS GBMs was further used for analysis using BRB-Array tools. Using class prediction module of BRB-Array tools^68^, mRNA levels of STS and LTS GBMs were compared and the expression changes with p-value less than 0.05 were considered significant. The list so obtained was then compared with quantitative proteomics data obtained in the current study.

Data arising from tissue proteomic analysis of the SVZ+, SVZ- GBM and normal brain tissue proteome was further subjected to bioinformatic analysis using the Database for Annotation, Visualization and Integrated Discovery (DAVID) version 6.7^69,70^ to identify the pathways affected in the sub groups of GBM patients.

Significantly altered proteins from the iTRAQ analysis of SVZ-, SVZ+ GBM tumors and peri-tumoral control tissues were subjected to partial least square discriminant analysis using METAGENassist, an online tool^71^. 243 common proteins arising from the iTRAQ based quantitative mass spectrometry data analysis were used for PLSDA and resulted in separation of SVZ- GBMs from SVZ+ GBMs as seen on the 3D-plot.

**ELISA**

Hemopexin levels in the GBM patient (both SVZ+ & SVZ-) serum samples was determined using competitive sandwich ELISA method (AssayPro ELISA kit, USA) following manufacturer’s instructions. Prior to performing the assay all the serum samples (n=20) were diluted (1:400) using diluent buffer. The diluted serum samples were added to the microplate coated with the polyclonal antibody against hemopexin and incubated for 1 hour. After 1 hour, the microplate wells were washed 5 times with 200 µl of wash buffer to remove the unbound components of the serum samples. 50 µl of Streptavidin-Peroxidase conjugate was added to each microplate well and incubated for 30 min followed by 5 washes with the wash buffer. Chromogenic substrate was added and incubated for 10 min followed by addition of stop solution to stop the reaction. The absorbance was read at two different wavelengths, i.e., 450 nm and 570 nm using SpectraMax microplate reader (Molecular devices). The absorbance values at 570 nm were substracted from the values at 450 nm and the resulting absorbance values were used for determining the serum concentrations of hemopexin from the standard curve.

**Western blotting**

Western blotting was performed on the serum samples of SVZ+ (n= 8) and SVZ- (n=8) GBM patients to validate the serum hemopexin levels.The serum proteins were separated using 12% SDS- polyacrylamide gels. 50 µg of serum protein samples were separated on the denaturing polyacrylamide gels were blotted on to PVDF membrane using semi-dry transfer unit (Amershan biosciences). Proper transfer of the protein bands on to the membrane was confirmed by ponceau S staining immediately after blotting. Overnight blocking was performed using 5% skimmed milk, followed by the addition of primary antibody (Rabbit polyclonal IgG, 1:500 dilution) against hemopexin (Santacruz biotechnology, lot no.J2909, sc-13443) and incubation for one hour at room temperature. Following incubation, HRP conjugated secondary antibody (1:2500 dilution, Goat anti-Rabbit IgG-HRP, GeNei, lot no. 032071) against the primary antibody was added to the membrane and incubated for one hour at room temperature. Once the incubation was over, the membrane was washed with TBST buffer. This washing step was performed thrice to remove unbound antibody, if any. A chromogenic substrate (TMB/H_2_O_2_, GeNei, lot no. 033091) was added to develop colour, followed by scanning the membrane using LabScan (GEhealthcare). Densitometric analysis of the hemopexin bands was performed using ImageQuant TL software (GE Healthcare).
